# Tanshinone IIA improves diabetes‐induced renal fibrosis by regulating the miR‐34‐5p/Notch1 axis

**DOI:** 10.1002/fsn3.2998

**Published:** 2022-08-05

**Authors:** Lizhi Zhang, Fan Yang

**Affiliations:** ^1^ Department of Nephrology The Second People's Hospital of Hefei, Medical University of Anhui (Hefei Hospital Affiliated to Medical University of Anhui) Hefei P.R. China; ^2^ Department of Nephrology The First Affiliated Hospital of Dalian Medical University Dalian P.R. China

**Keywords:** microRNA‐34a‐5p, notch receptor 1, renal fibrosis, tanshinone IIA

## Abstract

The purpose of this study was to evaluate the improvement of tanshinone in renal fibrosis in vitro and in vivo study. It used streptozotocin to model diabetic nephropathy (DN) mice, and treated with different Tanshinone IIA concentrations. The pathology of kidney tissues was evaluated by hematoxylin and eosin (H&E) and Masson's staining; the ultrastructure and apoptosis cell number of kidney tissues were evaluated by transmission electron microscopy (TEM) and TUNEL assay. Relative gene and protein expression was evaluated by reverse transcription‐quantitative polymerase chain reaction (RT‐qPCR), immunohistochemical (IHC) analysis, or western blot (WB) assay. In vitro study, using high‐glucose stimulated HK‐2 cell to model DN cell model, measuring cell proliferation, apoptosis rate, relative gene and protein expression, and LC 3B and P62 proteins expression by Cell Counting Kit‐8 (CCK‐8), flow cytometry, RT‐qPCR, WB, and cell immunofluorescence. Analysis correlation between Notch1 and miRNA‐34a‐5p was carried out by dual‐luciferase reporter. Fibrosis area and apoptosis cell rate were significantly up‐regulated (*p* < .001), with Tanshinone IIA supplement. The fibrosis area and apoptosis cell rate were also significantly improved in a dose‐dependent manner (*p* < .05). With si‐miRNA‐34a‐5p transfection, the Tanshinone IIA's treatment effects were significantly depressed. By dual‐luciferase reporter, miRNA‐34a‐5p could target Notch1 in the HK‐2 cell line. Tanshinone IIA improved DN‐induced renal fibrosis by regulating miRNA‐34a‐5p in vitro and in vivo study.

## Graphical abstract

Tanshinone IIA improved diabetic nephropathy (DN)‐induced renal fibrosis by regulating miRNA‐34a‐5p in vitro and in vivo study.

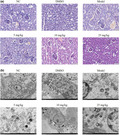

## 1 INTRODUCTION

Diabetic nephropathy (DN) is a diabetes mellitus (DM)‐induced renal microvascular disease, and is one of the most common complications of DM. It has been documented that 20%–40% of patients with DM also exhibit DN (Sharaf El Din et al., 2017). The pathological characteristics of DN include glomerular hypertrophy, glomerular basement membrane thickening, and mesangial matrix widening, which have the potential to progress into glomerular sclerosis and fibrosis. The pathogenesis of DN is complex, including nonenzymatic glycosylation and activation of the polyol and protein kinase C pathways, caused by disordered glucose metabolism that further progresses into lipid metabolism disorders, changes in renal hemodynamics, an imbalance of inflammatory factors and oxidative stress, and genetic alternations (Gregg et al., 2020). In the development of DN, the Notch signaling pathway induces renal injury by regulating apoptosis of renal tubular epithelial cells (Yao et al., 2017). The Notch signaling pathway plays a key role in cell differentiation, proliferation, and autophagy, a biological process involving protein degradation in membrane vesicles. Autophagy maintains the stability of the intracellular environment and the integrity of cells by degrading damaged organelles, aging proteins, and other macromolecular substances (Mizushima et al., 2008). The results of a previous study indicated that the autophagy of renal tubular epithelial cells was inhibited in a high‐glucose environment in vitro, which weakened the ability of tubular epithelial cells to remove extracellular matrix, thus promoting the process of renal interstitial fibrosis (Huang et al., 2014).

Tanshinone IIA is a lipid‐soluble monomer derived from *Salvia miltiorrhiza*. It is commonly used in the adjuvant treatment of cardiovascular diseases and ischemic stroke in Traditional Chinese Medicine (Yang et al., 2020). MicroRNAs (miRNAs/miRs) are noncoding RNAs, ~22 nucleotides in length, that bind messenger RNA (mRNA) to block the expression of protein coding genes to regulate transcription levels (Gholikhani‐Darbroud, 2020; Glynn, 2020; van der Kwast et al., 2019). However, the role of miRNA in the occurrence and treatment of renal fibrosis is yet to be fully elucidated. The results of previous studies have demonstrated a role of miR‐34a‐5p in fibrosis (Disayabutr et al., 2016; Li et al., 2011); however, the exact role of miR‐34a‐5p in diabetic‐induced renal fibrosis is yet to be fully understood. Thus, the present study aimed to determine the therapeutic effects of tanshinone IIA on renal fibrosis in vivo, and to examine any potential changes in the expression levels of associated genes. Furthermore, the present study aimed to determine the significance of miRNA and the associated downstream genes in tanshinone IIA therapy using in vitro cell experiments.

## 2 MATERIALS AND METHODS

### 2.1 Experimental animals (Chen et al., 2019)

A total of 60 6‐week‐old Kunming male mice (weight, 15 ± 5 g) were purchased from Nanjing Medical University. Each mouse was intraperitoneally injected with streptozotocin (pH, 4.5; 50 mg/kg), three times every other day. A total of 50 μl blood samples were collected from the tail vein in 3 h, and a blood glucose meter was used to determine and maintain a blood glucose level of ≥20 moL/L, to confirm the establishment of the DN model for 3 months.

A total of 54 Kunming mice were randomly divided into six groups: (i) Model group without any treatment (*n* = 9); (ii) dimethyl sulfoxide (DMSO) group treated with l ml/10 g d DMSO solution by gavage (*n* = 9); (iii) negative control (NC) group treated with 0.1% normal saline by gavage (*n* = 9); (iv) 5 mg/kg group (*n* = 9); (v) 10 mg/kg group (*n* = 9); and (vi) 25 mg/kg group (*n* = 9). A total of 1 ml tanshinone IIA at concentrations of 5, 10, and 25 mg/kg were given via gavage, respectively, at the same time from the first day following successful DN modeling. After 30 days of treatment, collecting 24‐h urinary protein by metabolic cage, using biuret method to measure urine protein (UP), the mice were anesthetized with 1% pentobarbital sodium (40 mg/kg) for retro‐orbital blood collection and the pinch reflex was monitored to ensure full anesthesia. After 1 ml blood collection, the mice were sacrificed via cervical dislocation, using automatic biochemical analyzer to measure serum creatinine (Cre) and blood urea nitrogen (BUN), and the left renal tissues were collected for H&E staining, Masson's staining, immunohistochemical (IHC) analysis, and observation under the transmission electron microscope. This study was approved by the Ethics Committee of The Second People's Hospital of Hefei.

### 2.2 Cell line and cell culture

The HK‐2 cells purchased from The Cell Bank of Type Culture Collection of The Chinese Academy of Sciences were cultured in Dulbecco's Modified Eagle's Medium/Nutrient Mixture F‐12 (DMEM/F12) (Sigma, USA) (1:1) containing 10% fetal bovine serum (FBS) (Sigma, USA) and 100 kU/L (kilounits per liter) penicillin–streptomycin in a 5% CO_2_ incubator at 37°C.

### 2.3 Cell transfection

HK‐2 cells in a logarithmic growth phase were inoculated into a cell flask and transfected using Lipofectamine® 2000 (Invitrogen; Thermo Fisher Scientific, Inc.) according to the manufacturer's protocol at room temperature for 24 h. Small interfering (si)‐miR‐34a‐5p (5′‐CUACCU GCACCAACAGCACUU‐3′), si‐NC (negative control)(5′‐CAGUACUUUUGUGUAGUACAA‐3′), and miR‐34a‐3p (5′‐UGGCAGUGUCUUAGCUGGUUGU‐3′) (Nanjing KeyGen Biotech Co., Ltd.) were transfected into HK‐2 cells at a concentration of 50 nmoL/L. Subsequent experiments were conducted after 24 h of transfection.

### 2.4 Establishment of a renal fibrosis induced by high‐glucose cell model in vitro (Zhu et al., 2018)

Cells were inoculated in a six‐well plate until 60%–70% confluence was reached. The culture medium was replaced with serum‐free medium for a further 24 h of culture. HK‐2 cells were treated with 30 mM glucose to make renal fibrosis induced by the high‐glucose cell model. Following incubation, 0.1% normal saline was added to the NC and Model groups, DMSO was added to the DMSO group, and 5, 10, and 25 mg/L tanshinone IIA was added, respectively, for 30 min. Subsequently, cells in the NC and DMSO groups were treated with glucose at a concentration of 4.0 mmoL/L, while the 5 mg/L, 10 mg/L, and 25 mg/L groups were treated with high levels of glucose, at a concentration of 30 mM, to construct the HK‐2 DN cell model. Tan: The cells were treated with 25 mg/L Tan based on the DN cell model; miR‐34a‐5p: The cells which were transfected with miR‐34a‐5p were based on the DN cell model; Tan+si‐miRNA: The cells which were transfected with si‐miR‐34a‐5p were treated with 25 mg/L Tan based on the DN cell model; Tan+Notch 1 inhibitor: The cells were treated with 25 mg/L Tan and 10 μm Notch 1 inhibitor (Sigma, USA) based on the DN cell model; Tan+Autophagy inhibitor: The cells were treated with 25 mg/L Tan and 10 μm Autophagy inhibitor which were mammalian target of rapamycin (mTOR) agonists (Sigma, USA) based on the DN cell model.

### 2.5 Pathological examination of the kidney

Hematoxylin & eosin (H&E) (cat. KGA224, KeyGen, Nanjing, China) and Masson's (cat. KGMST‐8003, KeyGen, Nanjing, China) staining was performed to observe the pathological changes of renal tissue following left kidney tissue collection, according to the manufacturer's protocol. Five visual fields were randomly selected from each slice, and the collagen volume fraction (CVF) of mouse renal tissue was calculated with ImageJ software, and the average value was taken. CVF /% = collagen area/total visual field area × 100%.

### 2.6 Transmission electron microscopy

The kidneys of mice were collected, washed with normal saline, and dried using filter paper. The renal cortex was cut into blocks (~1 mm^3^) and soaked in 2.5% glutaraldehyde solution. Blocks were fixed overnight at 4°C and washed in phosphate‐buffered saline (PBS) three times. The processed tissues were subjected to Altmann staining, embedded in resin, and cut into slices ~70 nm in thickness. Tissues were placed on copper mesh and observed under a transmission electron microscope (×10,000) following re‐staining.

### 2.7 TUNEL assay

Renal tissues were prepared as paraffin‐embedded slices and apoptosis was detected using a TUNEL (terminal deoxynucleotidyl transferase dUTP nick end labeling) assay, according to the manufacturer's protocol (cat. KGA700, KeyGen, Nanjing, China). The apoptotic cells were defined as those with notable yellow brown granules or patches in the nucleus. In total, two sections were randomly selected from each mouse, and five visual fields (magnification, ×100) were selected from each section to count the number of apoptotic cells and total cells.

### 2.8 Immunohistochemical (IHC) staining

Following antigen repair and blocking, the slices were incubated with monoclonal antibodies against LC3B (cat. Ab232940, Abcam, UK) and P62 (cat. Ab185015, Abcam, UK) at the optimal dilutions at room temperature for 24 h. The sections were washed with PBS and incubated with antirabbit secondary antibody (cat. Ab150077, Abcam, UK) for 1 h. Following washing with PBS, the slices were incubated with avidin–biotin complex (ABC) reagent (cat. 64261, Abcam, UK) at room temperature for 30 min, followed by 3,3′‐diaminobenzidine development and hematoxylin re‐staining.

### 2.9 Western blot analysis

The HK‐2 cells or kidney tissues were lysed on ice using radio‐immunoprecipitation assay (RIPA) lysis buffer with 1% phenylmethylsulfonyl fluoride (Nanjing KeyGen Biotech Co., Ltd.) and then centrifuged at 12,000× *g* for 15 min at 4°C. Total protein was collected and then the concentration of the samples was quantified using a bicinchoninic acid (BCA) assay (Nanjing KeyGen Biotech Co., Ltd.). Next, the proteins (30 μg) were separated using a 10% SDS‐PAGE (sodium dodecyl sulfate‐polyacrylamide gel electrophoresis) and then transferred to polyvinylidene difluoride (PVDF) membranes using wet transfer. After incubation with 5% skimmed milk for 2 h at room temperature, the membranes were incubated with the corresponding primary antibodies such as rabbit antinotch receptor 1 (Notch1; 1:1000), PTEN (phosphatase and tensin homolog) (1:1000), LC3 (1:1000), P62 (1:1000), LC 3B (1:1000), Beclin1 (1:1000), ATG7 (autophagy related 7) (1:1000), and GAPDH (glyceraldehyde 3‐phosphate dehydrogenase) (1:4000) overnight at 4°C. The membranes were repeatedly washed with Tris‐buffered saline (TBS) containing 0.1% Tween 20 (TBS‐T) 3 times, washed once with TBS, and incubated with the secondary antibody (cat no. 7076; 1:500 dilution; Cell Signaling Technology, Inc.) at room temperature for 1 h. Protein bands were visualized using the Clarity™ Western ECL substrate (Bio‐Rad Laboratories, Inc.). The protein expression was semiquantified using ImageJ software (Version 1.8.0; National Institutes of Health).

### 2.10 Reverse transcription‐quantitative (RT‐qPCR)

Total RNA was extracted from cells and kidney tissues using the one‐step method, according to the TRIzol® kit instructions (Invitrogen; Thermo Fisher Scientific, Inc.). The absorbance (A) values of RNA at 260 and 280 nm were measured using a nucleic acid spectrometer to maintain an A260/A280 ratio of 1.8–2.2. Based on the A value of 260 nm, the total RNA of the sample was quantified. Subsequently, the complementary DNA (cDNA) was synthesized using a reverse transcription kit (Invitrogen; Thermo Fisher Scientific, Inc.) (30°C 10 min, 42°C30 min, 99°C5 min, 4°C 5 min). Primer sequences are displayed in Table 1. The expression levels of miR‐34a‐5p, Notch1, PTEN, AKT, and mTOR were detected via fluorescence used in qPCR. The expression of miR‐34a‐5p was standardized with U6 mRNA, and those of Notch1, PTEN, AKT, and mTOR were normalized with GAPDH mRNA. Relative primer sequence is shown in Table 1.

**TABLE 1:** The primer sequence

Gene name	F:(5′‐3′)	R:(5′‐3′)
miRNA‐34a‐5p	ACACTCCAGCTGGGTGGCAGTGTCTTAGCT	TGGTGTCGTGGAGTCG
PTEN	GGTCTGAGTCGCCTGTCACCAT	CCGTGTTGGAGGCAGTAGAAGG
Notch1	AGCAAGGAGGCCAAGGACC	GACCCGCCCACAGTGAAA
AKT	CAGGATGTGGACCAACGTGAGG	GGCAGCGGATGATGAAGGTGTT
mTOR	CGTCAGCACCATCAACCTCCAA	TCAGCCGTCTCAGCCATTCCA
U6	CTCGCTTCGGCAGCACA	AACGCTTCACGAATTTGCGT
GAPDH	ACAGCAACAGGGTGG TGGAC	TGAGTCCTTCCACGATACCAA

### 2.11 Cell counting kit‐8 (CCK‐8) detection

Cells in each group were treated by different methods in different groups for 24 h, followed by the addition of 10 μl CCK‐8 reagent into each well for 2 h. The optical density was measured at a wavelength of 450 nm for the calculation of cell proliferation rate in each group. In total, three replicates were performed for each group and the experiment was repeated at least three times.

### 2.12 Flow cytometry for the detection of cell apoptosis

Apoptosis of cells in each group was detected using Annexin V‐FITC (fluorescein isothiocyanate) and propidium iodide (PI) dual staining. Following 24 h of treatment for each group, the adherent cells were digested with ethylenediaminetetra acid (EDTA)‐free trypsin and centrifuged at 4°C and 8000× *g* for 5 min. Subsequently, 5 × 10^5^ cells were collected and resuspended in 100 μl of 1X binding buffer, followed by the addition of 5 μl Annexin V‐FITC and 5 μl PI staining solution. Following incubation for 10 min in the dark at room temperature, 400 μl 1X binding buffer was added, and flow cytometry was carried out within 1 h.

### 2.13 Cell immunofluorescence assay

Following 24 h of treatment, cells in each group were washed three times in precooled PBS. Cells were fixed with 4% paraformaldehyde on ice for 15 min, washed three times with TBS‐T with shaking for 5 min, incubated with 0.3% Triton‐100 permeating treatment for 15 min, and washed three times with TBS‐T with shaking for 5 min. Cells were sealed with goat serum (Sigma, USA) for 30 min and incubated with the primary antibody including LC3B (cat. Ab232940, Abcam, UK, 1:1000) and P62 (cat. Ab185015, Abcam, UK, 1:1000) overnight at 4°C. Subsequently, cells were washed three times with TBS‐T with shaking for 5 min and incubated with the fluorescent secondary antibody (cat. ab6721, Abcam, UK, 1:500) in the dark for 1 h at room temperature. Cells were then stained with 0.1% DAPI (4′,6‐diamidino‐2‐phenylindole) staining for 6 min. Following a further three washing steps with TBS‐T for 5 min with shaking, sealing was performed with antifade mounting medium and images were captured using a fluorescence microscope (BX43, Olympus, Japan) in a dark room.

### 2.14 Dual‐luciferase reporter assay

The Notch1 3′‐untranslated region (UTR) was cloned into a pGL3 plasmid (cat. P19502, Shanghai Hewu Biotechnology Co., Ltd, Shanghai, China) to construct wild‐type and mutant Notch1 vectors. HK‐2 cells were inoculated in 12‐well plates and co‐transfected with wild‐type or mutant Notch1 vectors and the miR‐34a‐5p mimic (5′‐GAUGGACGUGCUUGUCGUGAAAC‐3′) and its control (miR‐NC; 5′‐UUCUCCGAACGUGUCACGUTT‐3′) using Lipofectamine 2000 (Thermo Fisher Scientific, Waltham, MA, USA). After 24 h, the firefly luciferase activity was detected using a Synergy H1 multifunctional Microplate Reader according to the instructions of the dual‐luciferase reporter gene assay kit (Abnova, Taipei, Taiwan). The activity of renilla luciferase was used as an internal reference. The wild‐type and mutant sequences of Notch1 3′‐UTR are shown in Table 2.

**TABLE 2:** The WT and Mut sequences of Nothch1

pmoGLO‐NOTCH1‐WT: ACGGCGCGCCCCACGAGACCCCGGCTTCCTTTCCCAAGCCTTCGGGCGTCTGTGTGCGCTCTGTGGATGCCAGGGCCGACCAGAGGAGCCTTTTTAAAACACATGTTTTTATACAAAATAAGAACGAGGATTTTAATTTTTTTTAGTATTTATTTATGTACTTTTATTTTACACAGAAACACTGCCTTTTTATTTATATGTACTGTTTTATCTGGCCCCAGGTAGAAACTTTTATCTATTCTGAGAAAACAAGCAAGTTCTGAGAGCCAGGGTTTTCCTACGTAGGATGAAAAGATTCTTCTGTGTTTATAAAATATAAACAAAGATTCATGATTTATAAATGCCATTTATTTATTGATTCCTTTTTTCAAAATCCAAAAAGAAATGATGTTGGAGAAGGGAAGTTGAACGAGCATAGTCCAAAAAG
pmoGLO‐NOTCH1‐MUT: ACGGCGCGCCCCACGAGACCCCGGCTTCCTTTCCCAAGCCTTCGGGCGTCTGTGTGCGCTCTGTGGATGCCAGGGCCGACCAGAGGAGCCTTTTTAAAACACATGTTTTTATACAAAATAAGAACGAGGATTTTAATTTTTTTTAGTATTTATTTATGTACTTTTATTTTACACAGAAAACAGTATTTTTTATTTATATGTACTGTTTTATCTGGCCCCAGGTAGAAACTTTTATCTATTCTGAGAAAACAAGCAAGTTCTGAGAGCCAGGGTTTTCCTACGTAGGATGAAAAGATTCTTCTGTGTTTATAAAATATAAACAAAGATTCATGATTTATAAATGCCATTTATTTATTGATTCCTTTTTTCAAAATCCAAAAAGAAATGATGTTGGAGAAGGGAAGTTGAACGAGCATAGTCCAAAAAG

### 2.15 Statistical analysis

The experimental data were analyzed using GraphPad Prism 6 software (GraphPad Software, Inc.). All experiments were conducted at least three times, and the data are expressed as the mean ± SD. Under the condition of meeting normal distribution and homogeneity of variance, quantitative data between or among groups were analyzed using two‐paired‐tailed t‐tests and one‐way analysis of variance (ANOVA) with Tukey's post hoc test. A value of *p* < .05 was considered to indicate a statistically significant difference.

## 3 RESULTS

### 3.1 Tanshinone IIA causes histopathological and ultrastructural changes in the kidney

There were no significant differences between NC and DMSO groups in 24‐h urinary protein, Cre and BUN levels (*p* > .05, Table 3). Compared with the NC group, 24‐h urinary protein, Cre and BUN levels were significantly up‐regulated in Model group (*p* < .001, respectively, Table 3); with Tanshinone IIA supplement, compared with Model group, 24‐h urinary protein, Cre and BUN levels were significantly improved in 5, 10, and 25 mg/kg groups (*p* < .05, respectively, Table 3) and there were significant differences among 5, 10, and 25 mg/kg groups in 24‐h urinary protein, Cre and BUN levels (*p* < .05, respectively, Table 3).

**TABLE 3:** Renal function in different groups (*n* = 9, mean ± SD)

	24‐h urinary protein (μmol/L)	Cre (μmol/L)	BUN (mmol/L)
NC	32.15 ± 2.64	12.87 ± 0.58	7.45 ± 1.14
DMSO	31.47 ± 2.61	13.02 ± 0.69	7.77 ± 1.82
Model	632.92 ± 62.88***	29.88 ± 1.52***	22.37 ± 0.82***
5 mg/kg	458.01 ± 23.25^#^	29.28 ± 2.57^#^	20.41 ± 1.10^#^
10 mg/kg	308.08 ± 34.46^##^ ^,^ ^$^	25.81 ± 1.96^##^ ^,^ ^$^	16.41 ± 0.89^##^ ^,^ ^$^
25 mg/kg	148.11 ± 21.32^###^ ^,^ ^$$^ ^,^ ^&^	16.53 ± 2.06^###^ ^,^ ^$$^ ^,^ ^&^	9.48 ± 0.88^###^ ^,^ ^$$^ ^,^ ^&^

*** *p* < .001, vs. NC;

^#^ *p* < .05,

^##^ *p* < .01,

^###^ *p* < .001, vs. Model;

^$^ *p* < .05,

^$$^ *p* < .01, vs. 5 mg/kg;

^&^ *p* < .05, vs. 10 mg/kg.

**FIGURE 1:**
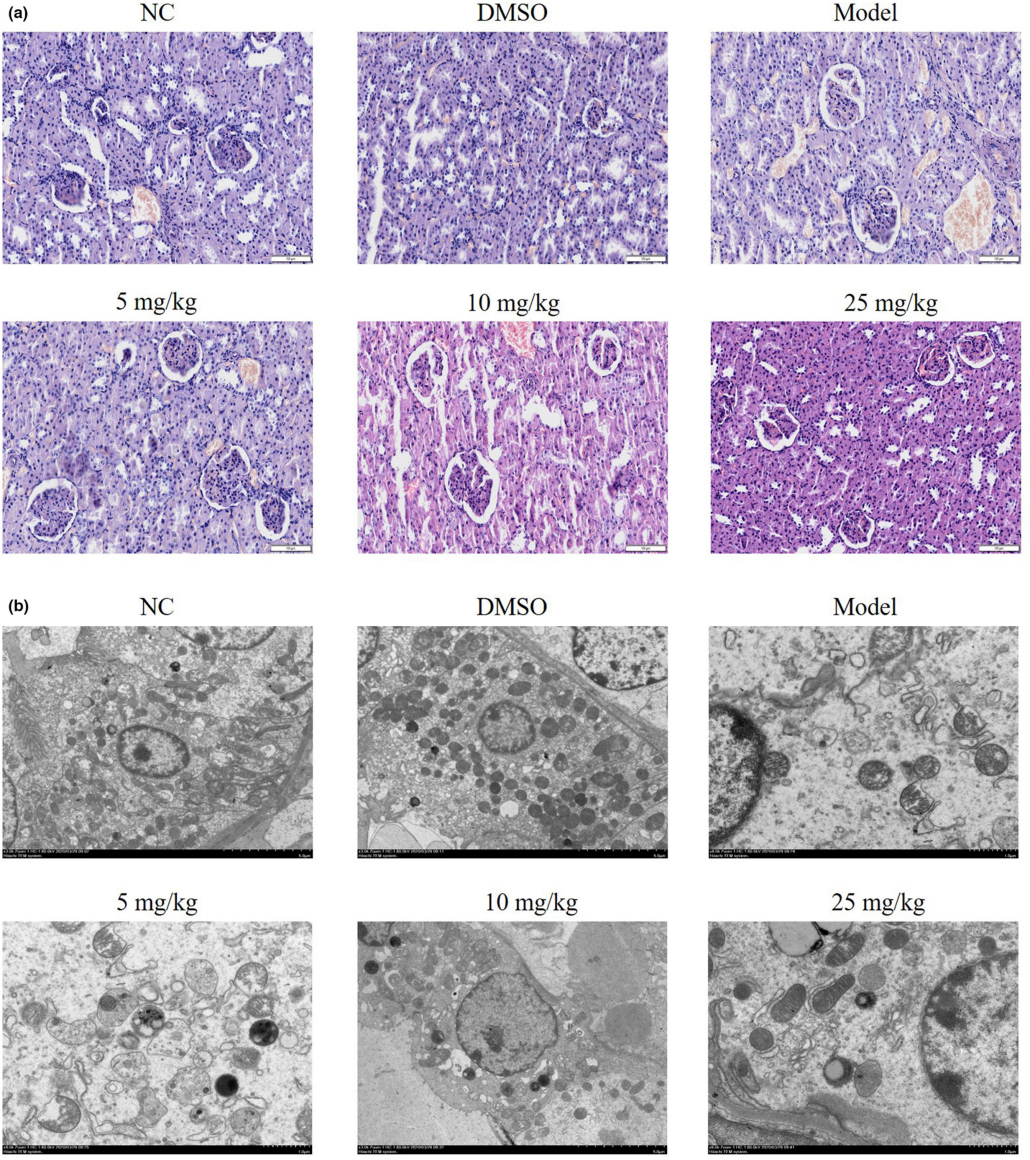
Histopathological and ultrastructural changes in the kidney. Mice were divided into the following groups: NC, mice treated with normal saline; DMSO, mice treated with DMSO; Model; DN mice; 5 mg/kg, DN mice treated with 5 mg/kg tanshinone IIA; 10 mg/kg, DN mice treated with 10 mg/kg tanshinone IIA; 25 mg/kg, DN mice treated with 25 mg/kg tanshinone IIA. (a) Histopathological analysis of kidney tissue using H&E staining. (b) Ultrastructural changes of kidney tissues were analyzed using transmission electron microscopy. DN, diabetic nephropathy; NC, negative control.

According to the results of the H&E staining demonstrated in Figure 1a, the tubular structure was clearly displayed in NC and DMSO groups, with no abnormality in the cell morphology and no interstitial edema. In the Model group, vacuolar degeneration of renal tubular epithelial cells, infiltration of inflammatory cells in the interstitium, and dilation of the renal tubules were apparent. Furthermore, following treatment with tanshinone IIA at varying concentrations, a significant improvement was observed in the vacuolar degeneration of renal tubular epithelial cells and infiltration of inflammatory cells in the interstitium.

Following the analysis of renal tissues using transmission electron microscopy (TEM), the Model group exhibited an increase in collagen fibers in the kidney tissue, a marked detachment of foot processes, the presence of fibrous lesions in the renal tubular lumen, and a decrease in autophagic bodies, compared with the NC group. Moreover, the 5 mg/kg, 10 mg/kg, and 25 mg/kg groups exhibited an increase in autophagic bodies, a decrease in epithelial cell exfoliation, and a decrease in the intraluminal lesions indicative of partial recovery, compared with the Model group (Figure 1b).

### 3.2 Tanshinone IIA affects fibrosis and apoptotic cells in renal tissue

**FIGURE 2:**
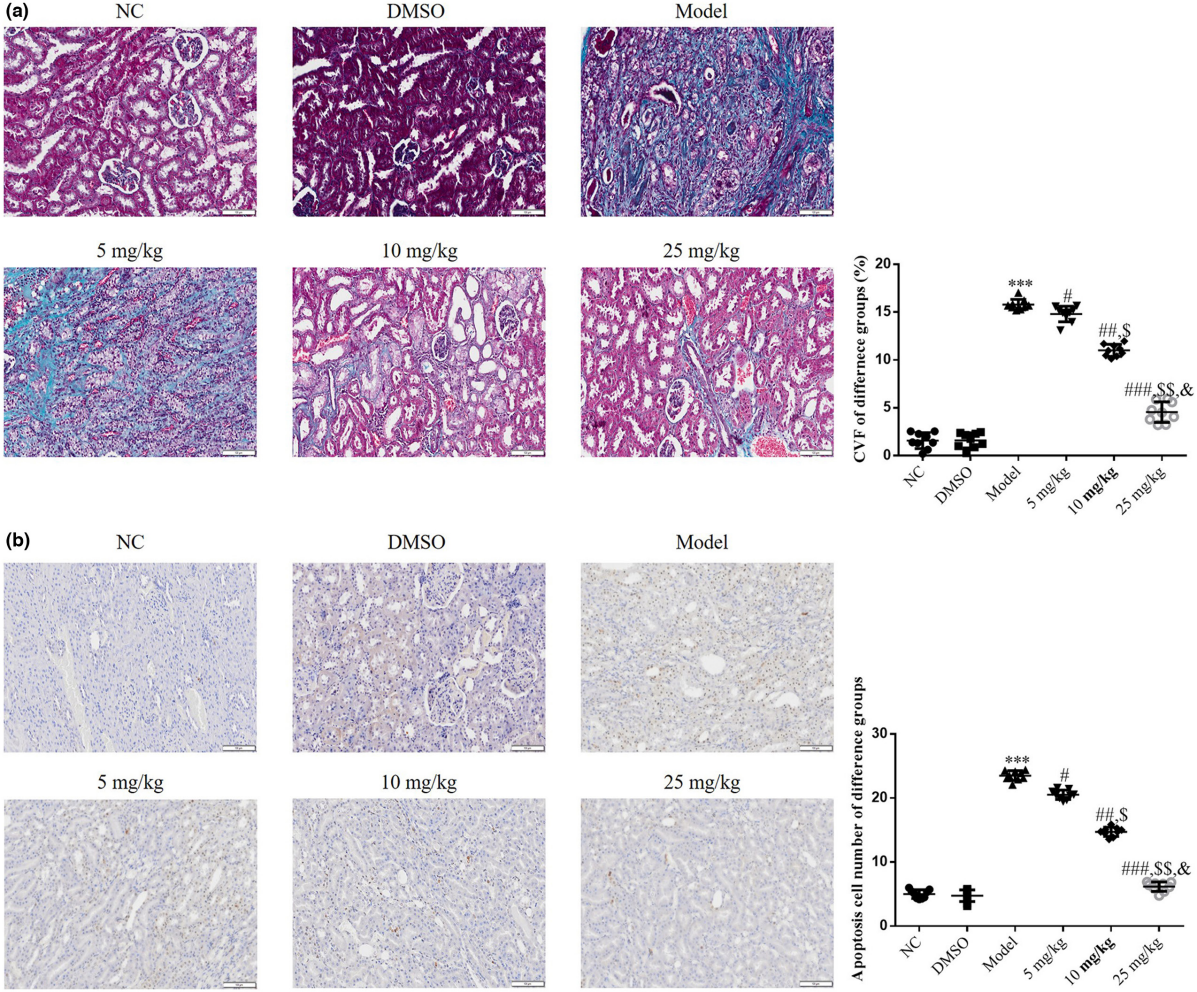
Fibrosis and apoptotic cells in renal tissue. Mice were divided into the following groups: NC, mice treated with normal saline; DMSO, mice treated with DMSO; Model; DN mice; 5 mg/kg, DN mice treated with 5 mg/kg tanshinone IIA; 10 mg/kg, DN mice treated with 10 mg/kg tanshinone IIA; 25 mg/kg, DN mice treated with 25 mg/kg tanshinone IIA. (a) Fibrosis level of different groups was observed using Masson's staining. (b) Apoptosis cell number of different groups was observed using the TUNEL assay. ***p < .001 vs. NC group; ^#^ *p* < .05, ^##^ *p* < .01, ^###^ *p* < .001 vs. Model group; ^$^ *p* < .05, ^$$^ *p* < .01 vs. 5 mg/kg group; ^&^ *p* < .05 vs. 10 mg/kg group. DN, diabetic nephropathy; NC, negative control.

As indicated by the results of Masson's staining, the collagen volume fraction (CVF) and the number of apoptotic cells were reduced in the NC and DMSO groups. Moreover, the Model group revealed significant increases in both the CVF and number of apoptotic cells (*p* < .001, respectively, Figure 2a,b), compared with the NC group. Following treatment with tanshinone IIA at different concentrations, the 5, 10, and 25 mg/kg groups exhibited an improvement in the CVF and the number of apoptotic cells in a dose‐dependent manner, compared with the Model group (*p* < .05, respectively, Figure 2a,b).

### 3.3 Tanshinone IIA impacts the expression levels of LC3B, P62, Beclin1, and ATG7 in renal tissue

**FIGURE 3:**
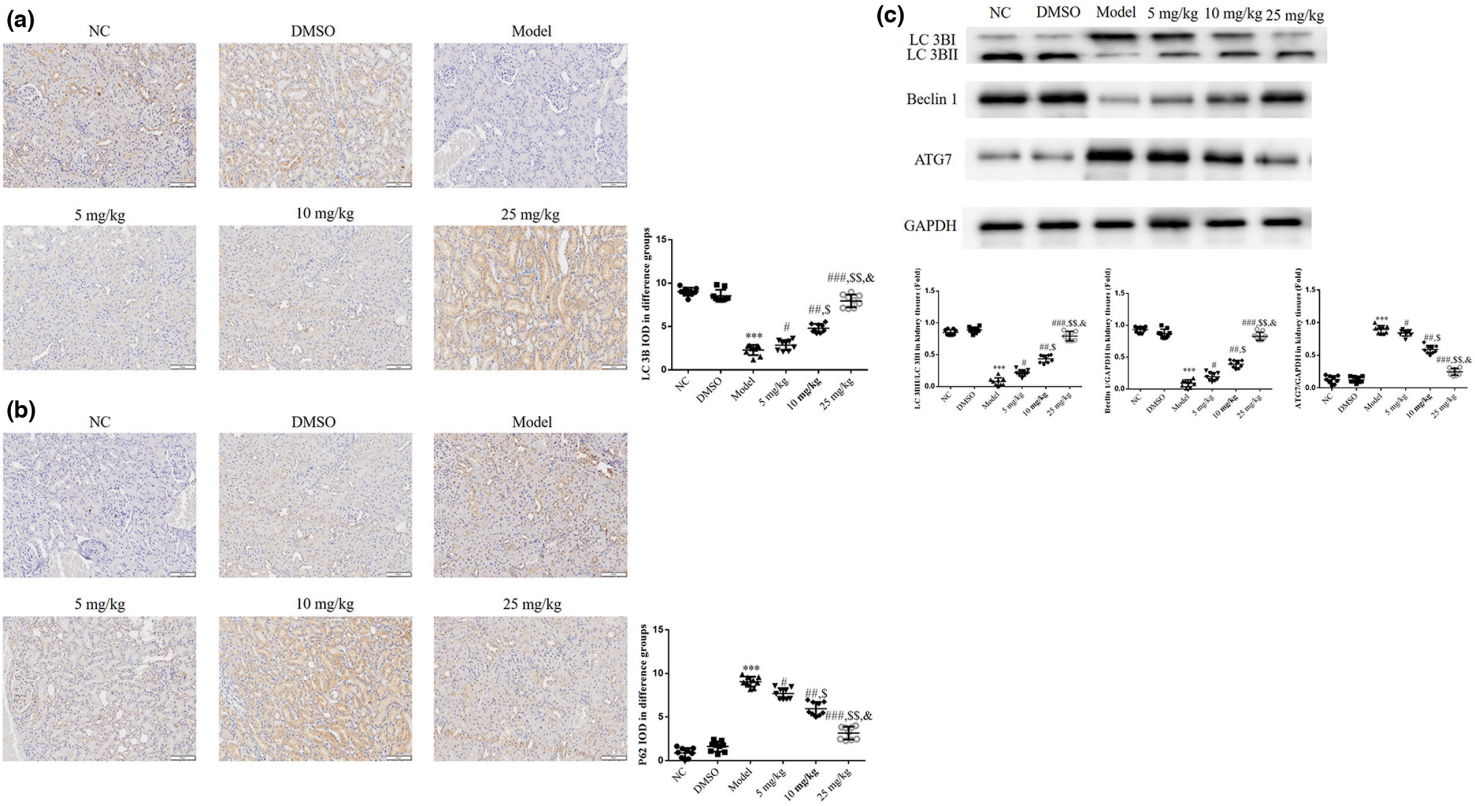
LC3B and P62 protein expression levels in kidney tissues analyzed using IHC staining. Mice were divided into the following groups: NC, mice treated with normal saline; DMSO, mice treated with DMSO; Model; DN mice; 5 mg/kg, DN mice treated with 5 mg/kg tanshinone IIA; 10 mg/kg, DN mice treated with 10 mg/kg tanshinone IIA; 25 mg/kg, DN mice treated with 25 mg/kg tanshinone IIA. (a) LC3B and (b) P62 protein expression in kidney tissues was analyzed using IHC staining. (c) LC 3BII/LC 3BI ratio, Beclin1 and ATG7 proteins by the western blot (WB) assay ^***^ *p* < .001 vs. NC group; ^#^ *p* < .05, ^##^ *p* < .01, ^###^ *p* < .001 vs. Model group; ^$^ *p* < .05, ^$$^ *p* < .01 vs. 5 mg/kg group; ^&^ *p* < .05 vs. 10 mg/kg group. DN, diabetic nephropathy; IHC, immunohistochemical; NC, negative control.

The results of IHC staining demonstrated a reduced level of LC3B protein expression and an increased level of P62 protein expression in the Model group (*p* < .001, respectively, Figure 3a,b), compared with the NC group. Following treatment with tanshinone IIA at varying concentrations, the levels of LC3B protein expression were increased, and the levels of P62 protein expression were decreased in the 5, 10, and 25 mg/kg groups in a dose‐dependent manner (*p* < .05, respectively, Figure 3a,b). By WB assay, compared with the NC group, LC 3BII/LC 3BI ratio, Beclin1 and ATG7 proteins were no significantly different in the DMSO group but were significantly changed in the Model group (*p* < .001, respectively, Figure 3c). Following treatment with tanshinone IIA at varying concentrations, compared with the Model group, LC 3BII/LC 3BI ratio and Beclin1 protein were significantly up‐regulated and ATG7 protein was significantly down‐regulated in 5 mg/kg, 10 mg/kg, and 25 mg/kg groups in a dose‐dependent manner (*p* < .05, respectively, Figure 3c).

### 3.4 Tanshinone IIA impacts the expression levels of associated proteins and genes

**FIGURE 4:**
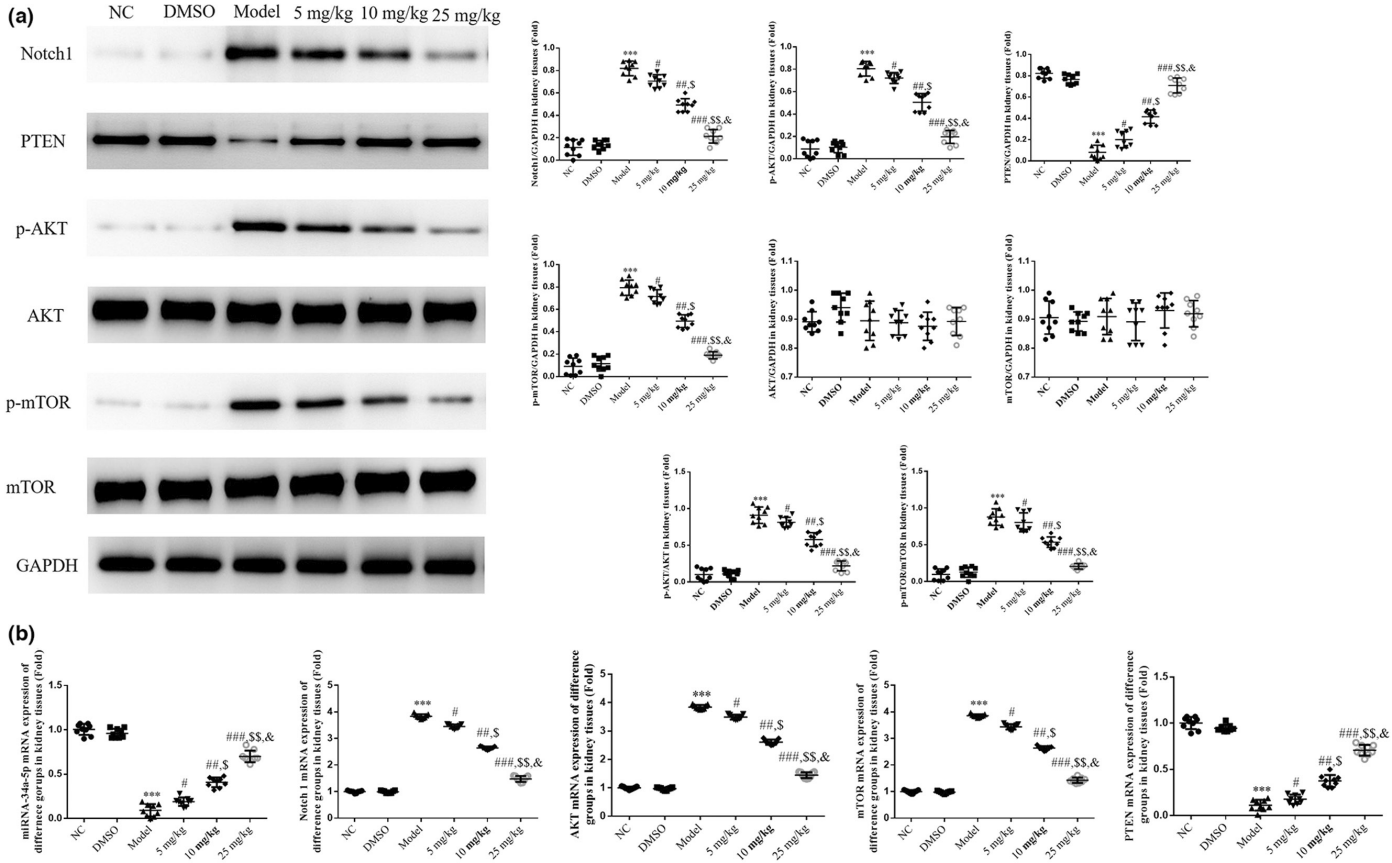
Relative protein and gene expression. Mice were divided into the following groups: NC, mice treated with normal saline; DMSO, mice treated with DMSO; Model; DN mice; 5 mg/kg, DN mice treated with 5 mg/kg tanshinone IIA; 10 mg/kg, DN mice treated with 10 mg/kg tanshinone IIA; 25 mg/kg, DN mice treated with 25 mg/kg tanshinone IIA. (a) Protein expression levels of Notch1, PTEN, p‐AKT, and p‐mTOR were analyzed using western blotting. (b) Gene expression levels of miR‐34a‐5p, Notch1, PTEN, AKT, and mTOR were analyzed using reverse transcription‐quantitative PCR. ^***^ *p* < .001 vs. NC group; ^#^ *p* < .05, ^##^ *p* < .01, ^###^ *p* < .001 vs. Model group; ^$^ *p* < .05, ^$$^ *p* < .01 vs. 5 mg/kg group; ^&^ *p* < .05 vs. 10 mg/kg group. DN, diabetic nephropathy; miR, microRNA; NC, negative control; Notch1, notch receptor 1; p‐, phosphorylated.

The results of the western blot analysis revealed a significant increase in the protein expression levels of Notch1, phosphorylated (p)‐AKT, and p‐mTOR, and a significant decrease in the protein expression level of PTEN in the Model group, compared with the NC group (*p* < .001, respectively, Figure 4a). Following treatment with tanshinone IIA at varying concentrations, the protein expression levels of Notch1, p‐AKT, and p‐mTOR were markedly decreased, while the protein expression level of PTEN was markedly increased in the 5, 10, and 25 mg/kg groups (*p* < .05, respectively, Figure 4a).

The results of RT‐qPCR analysis revealed an increase in the gene expression levels of Notch1, p‐AKT, and p‐mTOR, and a decrease in the gene expression levels of miR‐34a‐5p and PTEN in the Model group, compared with the NC group (*p* < .001, respectively, Figure 4b). Following treatment with tanshinone IIA at varying concentrations, the gene expression levels of Notch1, p‐AKT, and p‐mTOR were markedly reduced, and the gene expression levels of miR‐34a‐5p and PTEN were markedly increased in the 5, 10, and 25 mg/kg groups (*p* < .05, respectively, Figure 4b).

### 3.5 Tanshinone IIA impacts cell proliferation and apoptosis of high‐glucose‐induced HK‐2 cells

**FIGURE 5:**
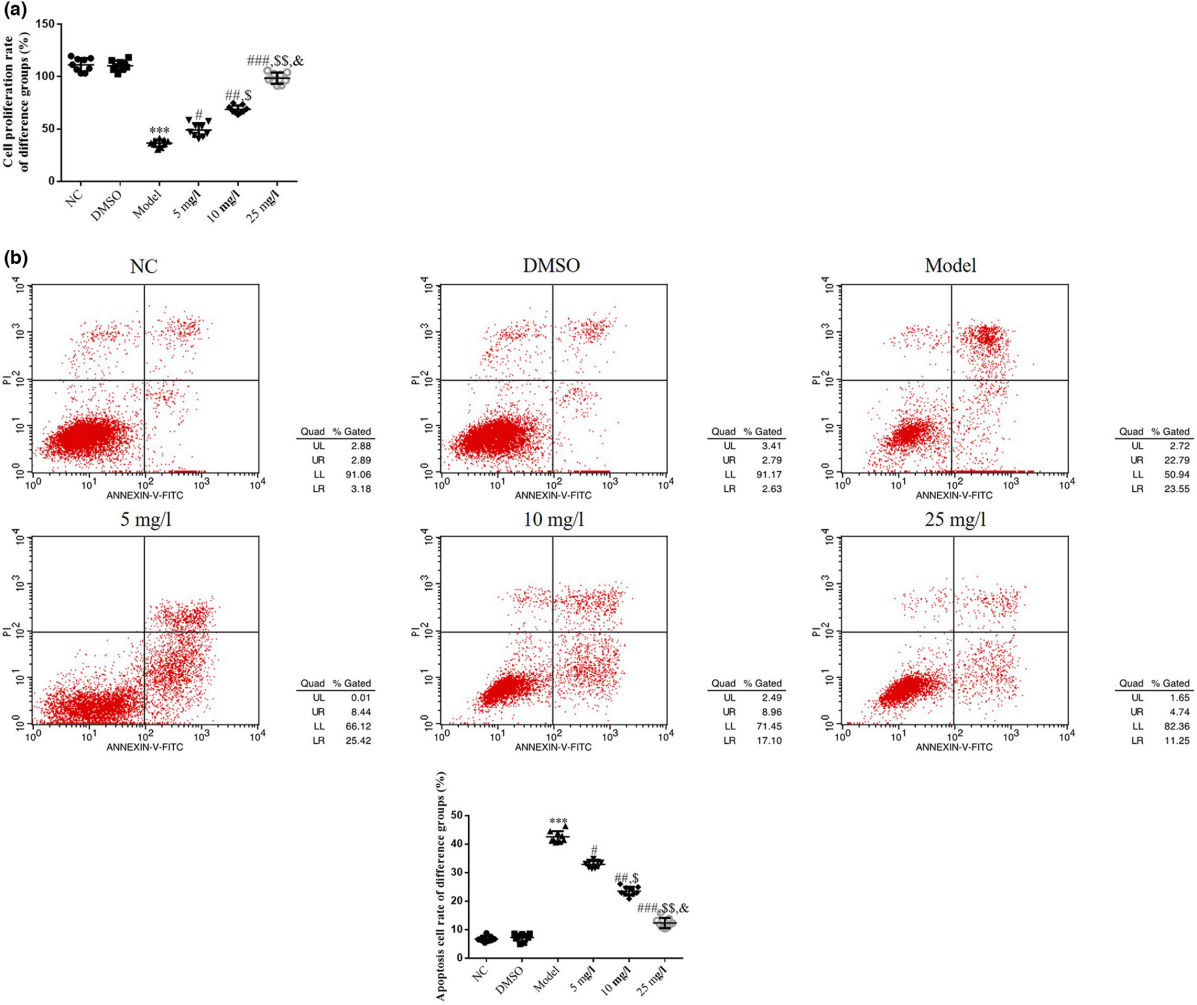
Effect of tanshinone IIA on proliferation and apoptosis of high‐glucose‐induced HK‐2 cells. The following groups were established: NC, HK‐2 cells treated with normal; DMSO: HK‐2 cells treated with DMSO; Model, HK‐2 cells treated with high concentrations of glucose; 5 mg/L, HK‐2 cells treated with 5 mg/L tanshinone IIA in high concentrations of glucose; 10 mg/L: HK‐2 cells treated with 10 mg/L tanshinone IIA in high concentrations of glucose; 25 mg/L, HK‐2 cells treated with 25 mg/L tanshinone IIA in high concentrations of glucose. (a) Cell proliferation rates analyzed using Cell Counting Kit‐8 assays. (b) Apoptosis rates of different groups. ^***^ *p* < .001 vs. NC group; ^#^ *p* < .05, ^##^ *p* < .01, ^###^ *p* < .001 vs. Model group; ^$^ *p* < .05, ^$$^ *p* < .01 vs. 5 mg/L group; ^&^ *p* < .05 vs. 10 mg/L group. DN, diabetic nephropathy; NC, negative control.

The results of the CCK‐8 assay indicated a significantly decreased rate of proliferation in the Model group, compared with the NC group (*p* < .001, respectively, Figure 5a). Following treatment with tanshinone IIA, the proliferation of HK‐2 cells in the 5, 10, and 25 mg/L groups was significantly improved in a dose‐dependent manner, compared with the Model group (*p* < .05, respectively, Figure 5a).

Moreover, the results of the flow cytometry analysis revealed an increase in cell apoptosis in the Model group compared with the NC group (*p* < .001, respectively, Figure 5b). Following treatment with tanshinone IIA in the 5, 10, and 25 mg/L groups, the cell apoptosis rate was significantly reduced in a dose‐dependent manner (*p* < .05, respectively, Figure 5b).

### 3.6 Effects of tanshinone IIA on associated protein expression levels

**FIGURE 6:**
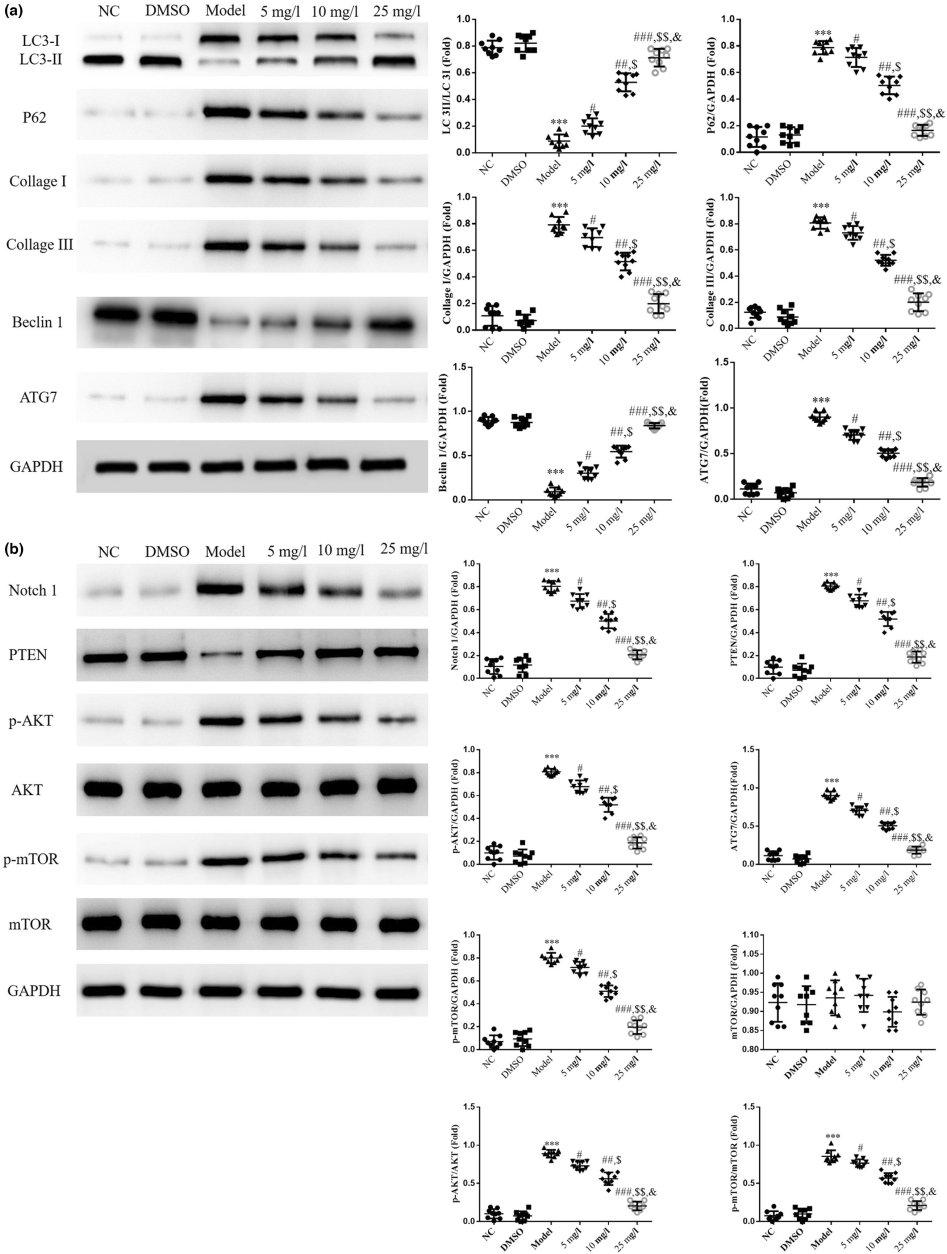
Relative proteins expression analyzed using western blotting. The following groups were established: NC, HK‐2 cells treated with normal; DMSO: HK‐2 cells treated with DMSO; Model, HK‐2 cells treated with high concentrations of glucose; 5 mg/L, HK‐2 cells treated with 5 mg/L tanshinone IIA in high concentrations of glucose; 10 mg/L: HK‐2 cells treated with 10 mg/L tanshinone IIA in high concentrations of glucose; 25 mg/L, HK‐2 cells treated with 25 mg/L tanshinone IIA in high concentrations of glucose. Protein expression levels of (a) LC3, P62, Beclin1, ATG7, Collagen I, and Collagen III and (b) Notch1, PTEN, p‐AKT, and p‐mTOR were detected via western blotting. ^***^ *p* < .001 vs. NC group; ^#^ *p* < .05, ^##^ *p* < .01, ^###^ *p* < .001 vs. Model group; ^$^ *p* < .05, ^$$^ *p* < .01 vs. 5 mg/L group; ^&^ *p* < .05 vs. 10 mg/L group. DN, diabetic nephropathy; NC, negative control; notch, notch receptor 1; p‐, phosphorylated.

The results of the western blot analysis revealed a decrease in the ratio of LC3II/LC3I and Beclin1 protein expression in the Model group, compared with the NC group (*p* < .001, Figure 6a). Moreover, protein expression levels of P62, ATG7, Collagen I, Collagen III, Notch1, PTEN, p‐AKT, and p‐mTOR were markedly increased in the Model group, compared with the NC group (*p* < .001, respectively, Figure 6a,b). Following treatment with tanshinone IIA, the ratio of LC3II/LC3I and Beclin1 protein was significantly increased, and the protein expression levels of P62, ATG7, Collagen I, Collagen III, Notch1, PTEN, p‐AKT, and p‐mTOR were significantly decreased in the 5, 10, and 25 mg/L groups in a dose‐dependent manner (*p* < .05, respectively, Figure 6a,b).

### 3.7 Effects of tanshinone IIA on associated genes

**FIGURE 7:**
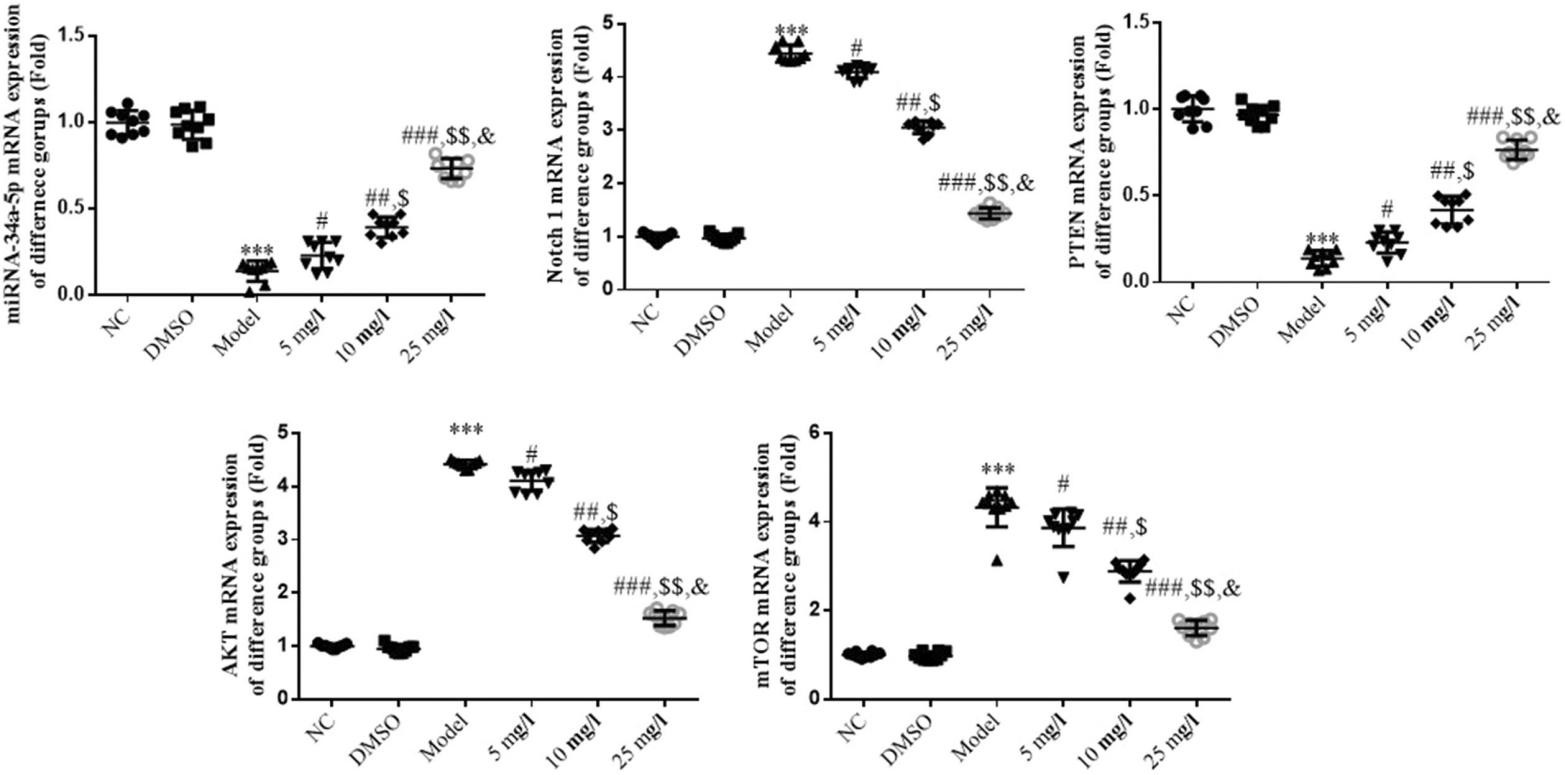
Relative gene expression in different groups determined using reverse transcription‐quantitative PCR analysis. The following groups were established: NC, HK‐2 cells treated with normal; DMSO: HK‐2 cells treated with DMSO; Model, HK‐2 cells treated with high concentrations of glucose; 5 mg/L, HK‐2 cells treated with 5 mg/L tanshinone IIA in high concentrations of glucose; 10 mg/L: HK‐2 cells treated with 10 mg/L tanshinone IIA in high concentrations of glucose; 25 mg/L, HK‐2 cells treated with 25 mg/L tanshinone IIA in high concentrations of glucose. ^***^ *p* < .001 vs. NC group; ^#^ *p* < .05, ^##^ *p* < .01, ^###^ *p* < .001 vs. Model group; ^$^ *p* < .05, ^$$^ *p* < .01 vs. 5 mg/L group; ^&^ *p* < .05 vs. 10 mg/L group. DN, diabetic nephropathy; NC, negative control.

The results of the RT‐qPCR analysis revealed a significant decrease in the gene expression levels of miR‐34a‐5p and PTEN, and a significant increase in the gene expression levels of Notch1, AKT, and mTOR in the Model group, compared with the NC group (*p* < .001, respectively, Figure 7). Moreover, following treatment with tanshinone IIA, a significant increase in the gene expression levels of miR‐34a‐5p and PTEN, and a significant decrease in the gene expression levels of Notch1, AKT, and mTOR were observed in the 5, 10, and 25 mg/L groups in a dose‐dependent manner, compared with the Model group (*p* < .05, respectively, Figure 7).

### 3.8 Effects of tanshinone IIA on LC3B and P62 protein expression levels

**FIGURE 8:**
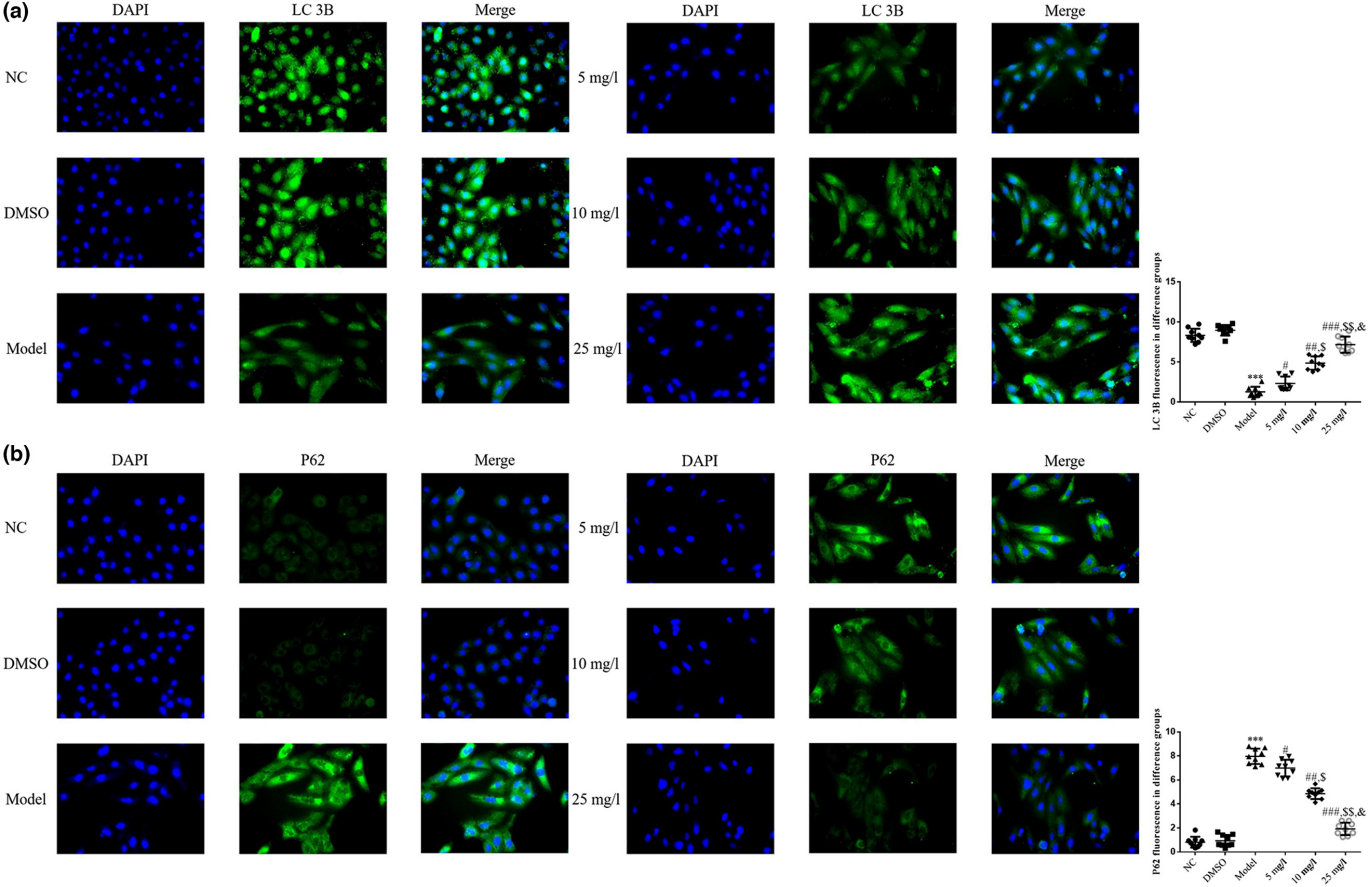
LC3B and P62 protein expression levels detected using cell immunofluorescence assays. The following groups were established: NC, HK‐2 cells treated with normal; DMSO: HK‐2 cells treated with DMSO; Model, HK‐2 cells treated with high concentrations of glucose; 5 mg/L, HK‐2 cells treated with 5 mg/L tanshinone IIA in high concentrations of glucose; 10 mg/L: HK‐2 cells treated with 10 mg/L tanshinone IIA in high concentrations of glucose; 25 mg/L, HK‐2 cells treated with 25 mg/L tanshinone IIA in high concentrations of glucose. (a) LC3B and (b) P62 protein expression levels detected by cell immunofluorescence assays (magnification, ×200). ^***^ *p* < .001 vs. NC group; ^#^ *p* < .05, ^##^ *p* < .01, ^###^ *p* < .001 vs. Model group; ^$^ *p* < .05, ^$$^ *p* < .01 vs. 5 mg/L group; ^&^ *p* < .05 vs. 10 mg/L group. DN, diabetic nephropathy; NC, negative control.

The results of the immunofluorescence staining analysis revealed a notable decrease in the protein expression level of LC3B, and a marked increase in the protein expression level of P62 in the Model group, compared with the NC group (*p* < .001, respectively, Figure 8a,b). Following treatment with tanshinone IIA, the protein expression level of LC3B was significantly increased, and the protein expression level of P62 was markedly decreased in the 5, 10, and 25 mg/L groups in a dose‐dependent manner (*p* < .05, respectively, Figure 8a,b).

### 3.9 Notch 1 inhibitor or Autophagy inhibitor effects in tanshinone IIA‐induced effects on HK‐2 cell proliferation and apoptosis

**FIGURE 9:**
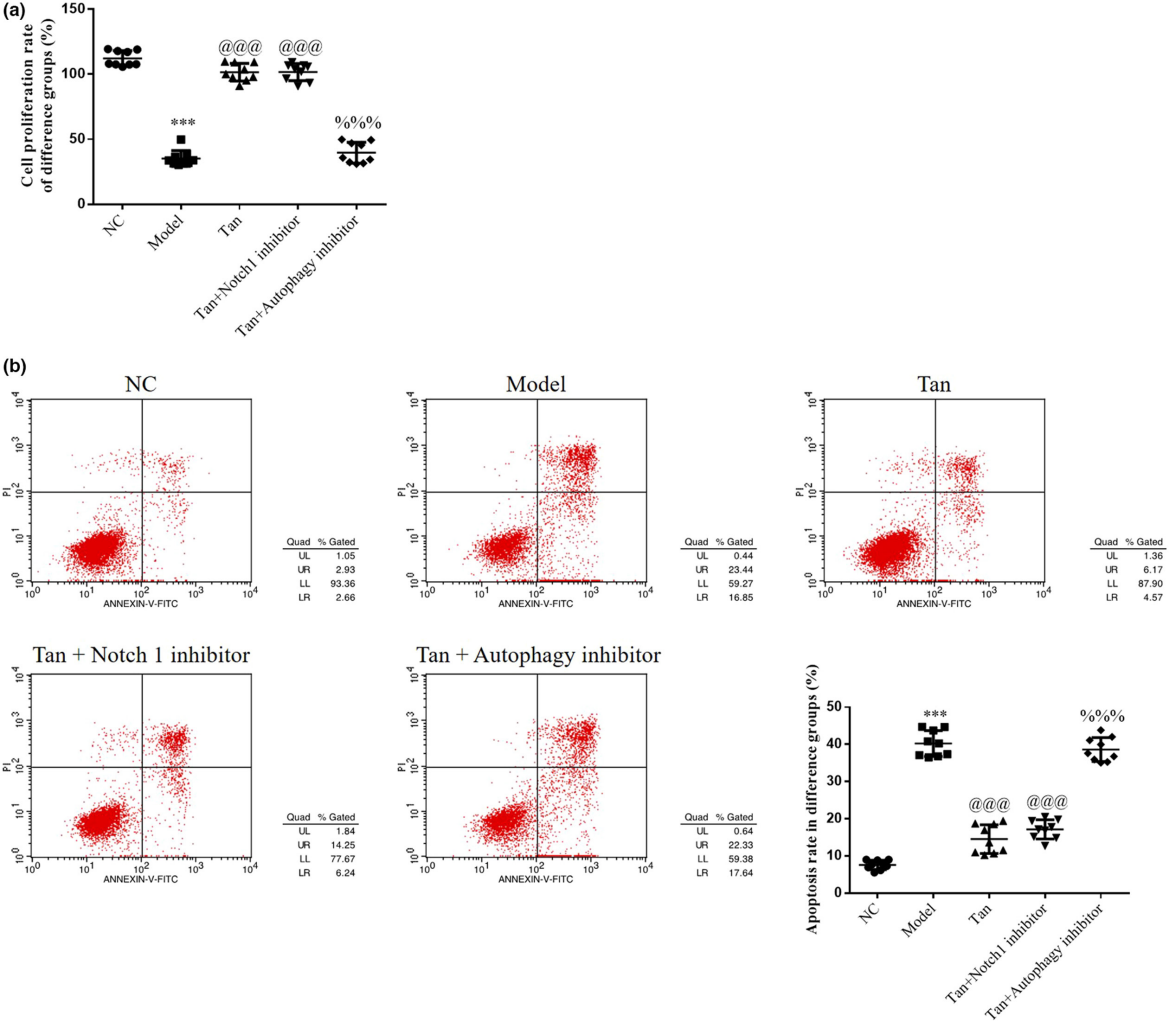
Notch 1 inhibitor or Autophagy inhibitor effects in tanshinone IIA‐induced effects on HK‐2 cell proliferation and apoptosis. The following groups were established: NC, HK‐2 cells treated with normal; Model, HK‐2 cells treated with high concentrations of glucose; Tan, HK‐2 cells treated with 25 mg/L tanshinone IIA in high concentrations of glucose; Tan+Notch 1 inhibitor: HK‐2 cells treated with 25 mg/L tanshinone IIA and 10 μm Notch 1 inhibitor in high concentrations of glucose; Tan+Autophagy inhibitor: HK‐2 cells treated with 25 mg/L tanshinone IIA and 10 μm Autophagy inhibitor in high concentrations of glucose.

As demonstrated in Figure 9a,b, a significantly reduced cell proliferation rate and an increased apoptosis rate were observed in the Model group (*p* < .001, respectively, Figure 9a,b), compared with the NC group. Moreover, an increase in the cell proliferation rate and a decrease in the apoptosis rate were observed in the Tan group (based on Model group treatment, the cells were treated with high‐dose Tan) following treatment with tanshinone IIA and Notch 1 inhibitor group (based on the Model group treatment, the cells were treated with 10 μm Notch 1 inhibitor) (*p* < .001, respectively, Figure 9a,b). A significant decrease in the rate of cell proliferation and a significant increase in the rate of cell apoptosis were observed in the Tan+Autophagy inhibitor group (based on the Model group treatment, the cells were treated with 10 μm Autophagy inhibitor) (*p* < .001, respectively, Figure 9a,b).

### 3.10 Notch 1 inhibitor or Autophagy inhibitor effects on associated proteins

**FIGURE 10:**
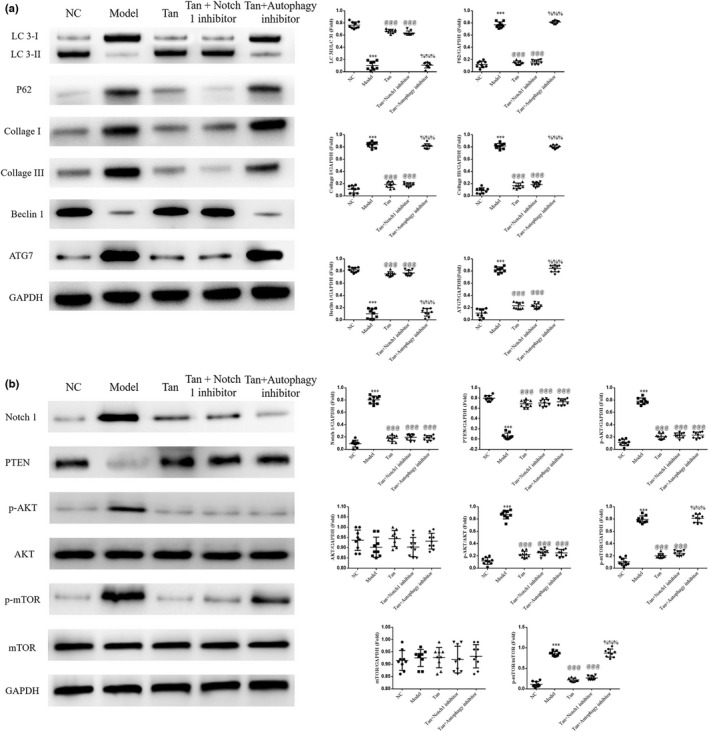
Notch 1 inhibitor or Autophagy inhibitor effects on associated proteins. The following groups were established: NC, HK‐2 cells treated with normal; Model, HK‐2 cells treated with high concentrations of glucose; Tan, HK‐2 cells treated with 25 mg/L tanshinone IIA in high concentrations of glucose; Tan+Notch 1 inhibitor: HK‐2 cells treated with 25 mg/L tanshinone IIA and 10 μm Notch 1 inhibitor in high concentrations of glucose; Tan+Autophagy inhibitor: HK‐2 cells treated with 25 mg/L tanshinone IIA and 10 μm Autophagy inhibitor in high concentrations of glucose. (a) Cell proliferation rate of different groups determined using Cell Counting Kit‐8 assays. (b) Apoptosis rate of different groups. ^***^ *p* < .001 vs. NC group; ^@@@^ *p* < .001 vs. Model group; ^%%%^ *p* < .001 vs. Tan group. NC, negative control.

The results of the western blot analysis revealed a decrease in the LC3II/LC3I ratio and Beclin1 protein and a significant increase in the protein expression levels of P62, ATG7, Collagen I, Collagen III, Notch1, PTEN, p‐AKT, and p‐mTOR in the Model group, compared with the NC group (*p* < .001, respectively, Figure 10a,b). Furthermore, following treatment with tanshinone IIA or/and Notch 1 inhibitor, the Tan and Tan+Notch 1 inhibitor groups demonstrated an increase in the LC3II/LC3I ratio and Beclin1 protein, and a marked decrease in the protein expression levels of P62, ATG7, Collagen I, Collagen III, Notch1, p‐AKT, and p‐mTOR, and PTEN was significantly up‐regulated (*p* < .001, respectively, Figure 10a,b). In the Tan+Autophagy inhibitor group, compared with the Tan group, LC3II/LC3I ratio, Beclin1, Notch1, and p‐AKT were significantly reduced, and P62, Collagen I, Collagen III, ATG7, and p‐mTOR proteins expression significantly increased (*p* < .001, respectively, Figure 10a,b). These results have shown that in the DN cell model, Tan and Notch 1 inhibitor also had the same treatment effects. Autophagy inhibitor showed effects to inhibit autophagy, however, the Autophagy inhibitor showed no effect to inhibit Notch1 and AKT, so these results may have caused the Autophagy inhibitor to be an mTOR agonist.

### 3.11 Notch 1 inhibitor or Autophagy inhibitor effects on relative mRNA expression

**FIGURE 11:**
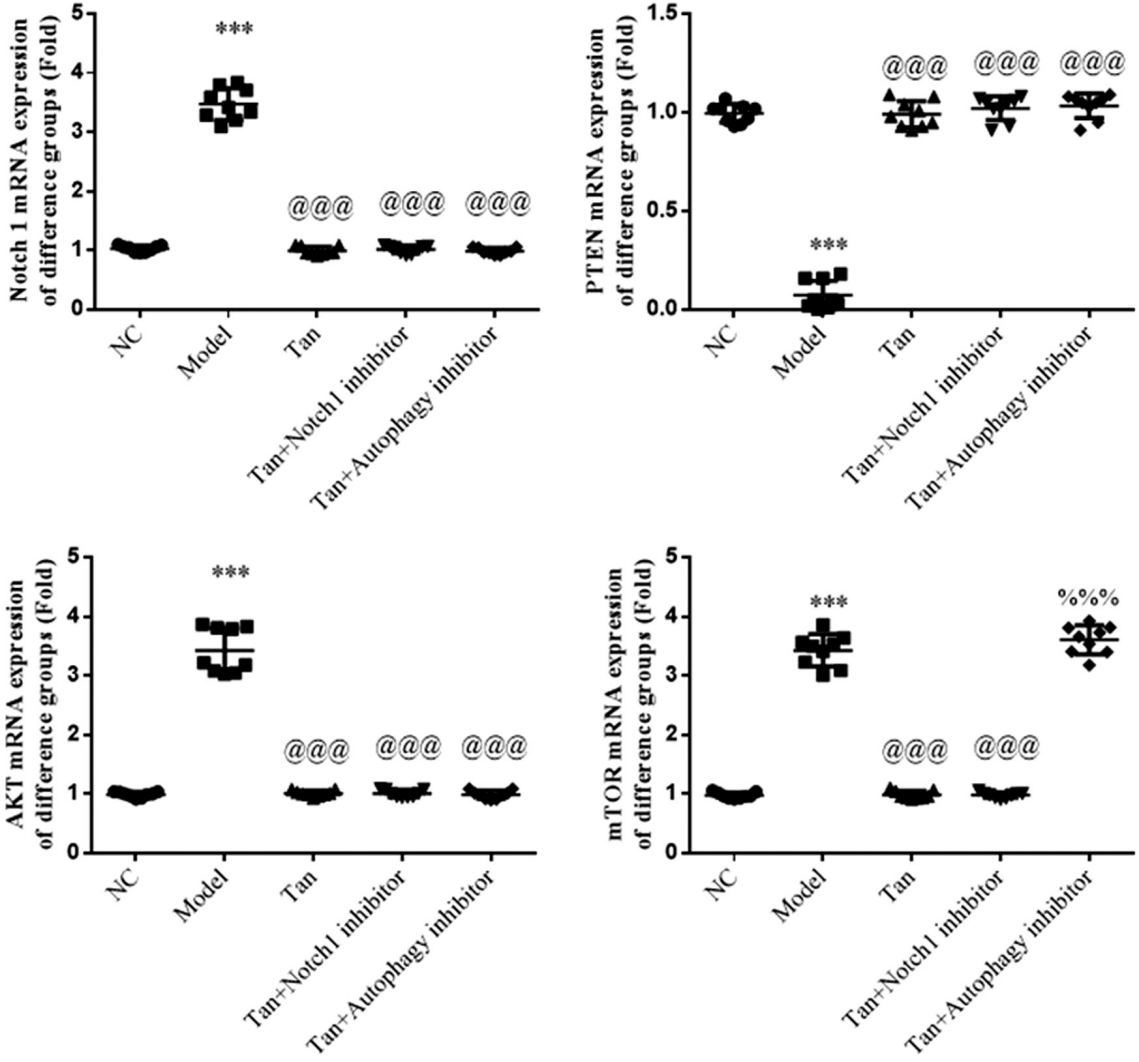
Notch 1 inhibitor or Autophagy inhibitor effects to relative mRNA expression. The following groups were established: NC, HK‐2 cells treated with normal; Model, HK‐2 cells treated with high concentrations of glucose; Tan, HK‐2 cells treated with 25 mg/L tanshinone IIA in high concentrations of glucose; Tan+Notch 1 inhibitor: HK‐2 cells treated with 25 mg/L tanshinone IIA and 10 μm Notch 1 inhibitor in high concentrations of glucose; Tan+Autophagy inhibitor: HK‐2 cells treated with 25 mg/L tanshinone IIA and 10 μm Autophagy inhibitor in high concentrations of glucose. Cell proliferation rate of different groups determined using Cell Counting Kit‐8 assays. Apoptosis rate of different groups. ^***^ *p* < .001 vs. NC group; ^@@@^ *p* < .001 vs. Model group; ^%%%^ *p* < .001 vs. Tan group. NC, negative control.

By the RT‐qPCR assay, compared with the NC group, Notch1, AKT, and mTOR mRNA expression was significantly up‐regulated and PTEN mRNA expression was significantly down‐regulated in the Model group (*p* < .001, respectively, Figure 11); compared with the Model group, Notch 1 and AKT mRNA expressions were significantly down‐regulated and the PTEN mRNA expression was significantly down‐regulated in Tan, Tan+Notch 1 inhibitor, and Tan+Autophagy inhibitor groups (*p* < .001, respectively, Figure 11) and mTOR mRNA expression was significantly down‐regulated in Tan and Tan+Notch 1 inhibitor groups (*p* < .001, respectively, Figure 11); compared with the Tan group, mTOR mRNA expression was significantly up‐regulated in the Tan+Autophagy inhibitor group (*p* < .001, Figure 11). These results have shown that autophagy inhibitor played suppressing autophagy effects via stimulating mTOR.

### 3.12 Notch 1 inhibitor or Autophagy inhibitor effects on the protein expression levels of LC3B and P62

**FIGURE 12:**
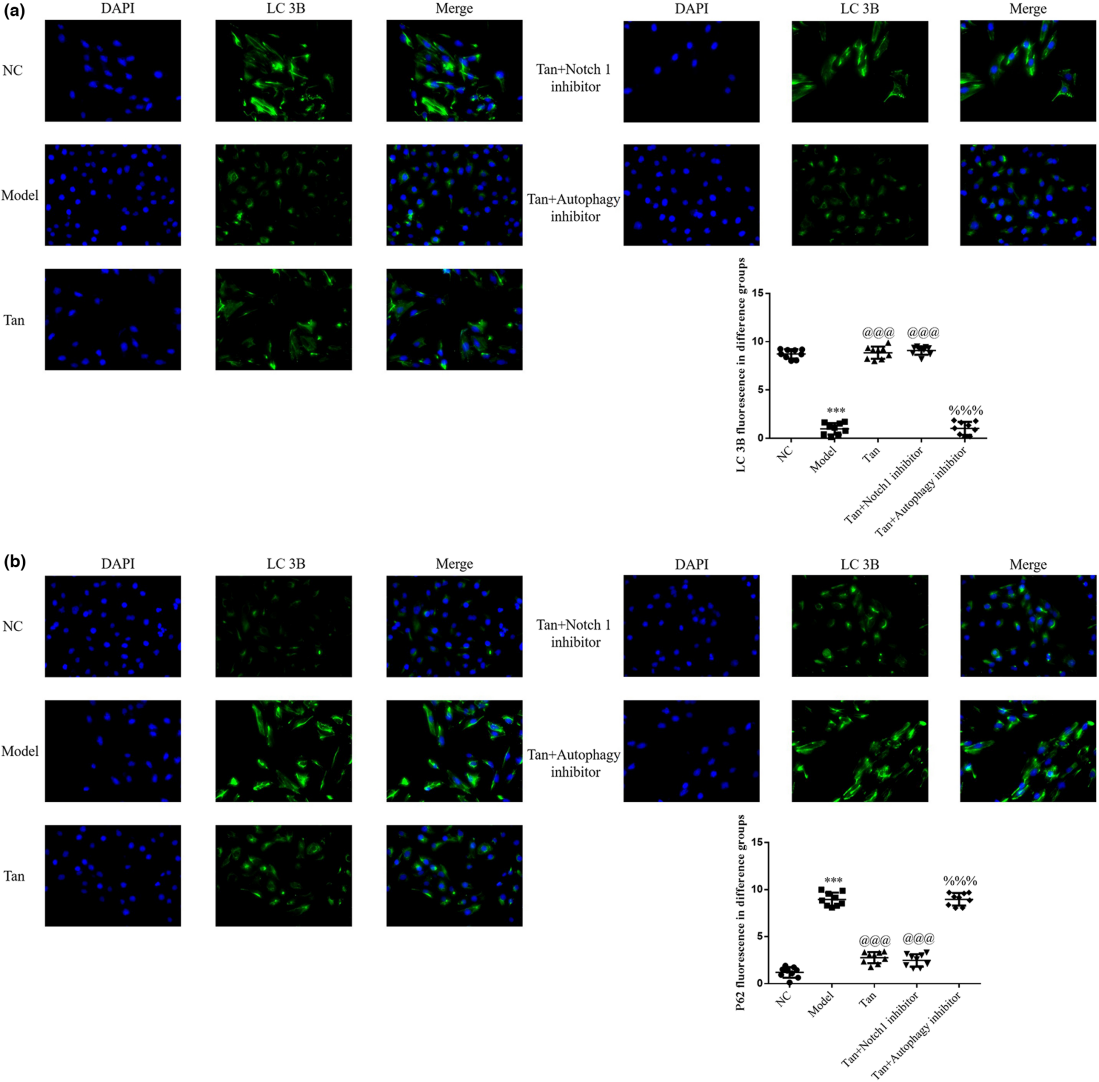
Notch 1 inhibitor or Autophagy inhibitor effects on the protein expression levels of LC3B and P62. The following groups were established: NC, HK‐2 cells treated with normal; Model, HK‐2 cells treated with high concentrations of glucose; Tan, HK‐2 cells treated with 25 mg/L tanshinone IIA in high concentrations of glucose; Tan+Notch 1 inhibitor: HK‐2 cells treated with 25 mg/L tanshinone IIA and 10 μm Notch 1 inhibitor in high concentrations of glucose; Tan+Autophagy inhibitor: HK‐2 cells treated with 25 mg/L tanshinone IIA and 10 μm Autophagy inhibitor in high concentrations of glucose. (a) Cell proliferation rate of different groups determined using Cell Counting Kit‐8 assays. (b) Apoptosis rate of different groups. ^***^ *p* < .001 vs. NC group; ^@@@^ *p* < .001 vs. Model group; ^%%%^ *p* < .001 vs. Tan group. NC, negative control.

The results of the immunofluorescence assay demonstrated a decrease in the protein expression level of LC3B, and an increase in the protein expression level of P62 in the Model group, compared with the NC group (*p* < .001, respectively, Figure 12a,b). Following treatment with tanshinone IIA or/and Notch 1 inhibitor, an increase in the protein expression level of LC3B and a significant decrease in the protein expression level of P62 were observed in the Tan and Tan+Notch 1 inhibitor groups (*p* < .001, respectively, Figure 12a,b). In addition, a decrease in the protein expression level of LC3B and an increase in the protein expression level of P62 were observed in the Tan+Autophagy inhibitor group compared with the Tan group (*p* < .001, respectively, Figure 12a,b).

### 3.13 Role of miR‐34a‐5p in tanshinone IIA‐induced effects on HK‐2 cell proliferation and apoptosis

**FIGURE 13:**
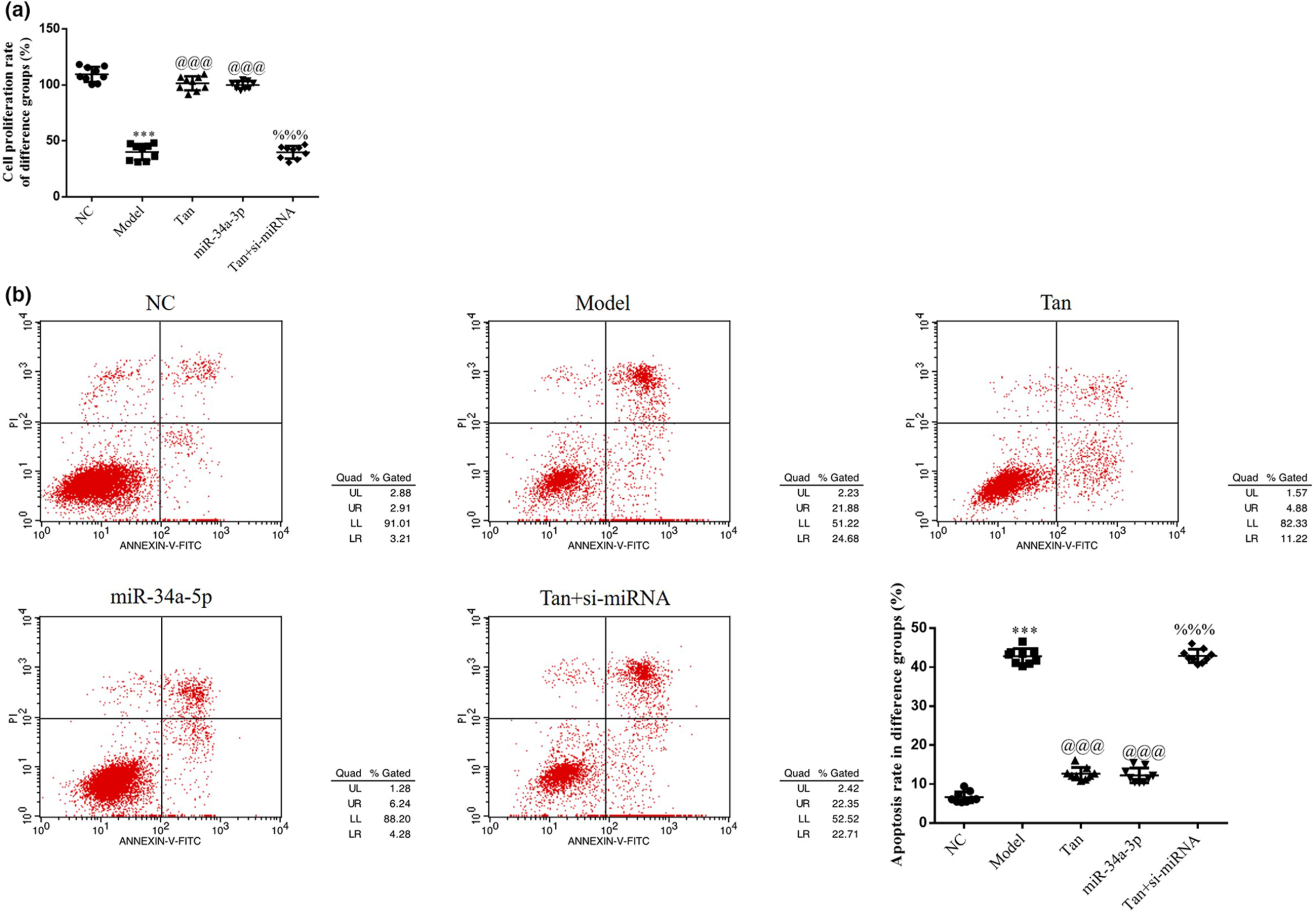
miR‐34a‐5p affects cell proliferation and apoptosis. The following groups were established: NC, HK‐2 cells treated with normal; Model, HK‐2 cells treated with high concentrations of glucose; Tan, HK‐2 cells treated with 25 mg/L tanshinone IIA in high concentrations of glucose; miR‐34a‐5p: HK‐2 cells transfected with miR‐34a‐5p mimics in high concentrations of glucose; Tan + si‐miRNA, HK‐2 cells transfected with si‐miR‐34a‐5p and treated with 25 mg/L tanshinone IIA in high concentrations of glucose. (a) Cell proliferation rate of different groups determined using Cell Counting Kit‐8 assays. (b) Apoptosis rate of different groups. ^***^ *p* < .001 vs. NC group; ^@@@^ *p* < .001 vs. Model group; ^%%%^ *p* < .001 vs. Tan group. miRNA/miR, microRNA; NC, negative control.

As demonstrated in Figure 13a,b, a significantly reduced cell proliferation rate and an increased apoptosis rate were observed in the Model group (*p* < .001, respectively, Figure 13a,b), compared with the NC group. Moreover, an increase in the cell proliferation rate and a decrease in the apoptosis rate were observed in the Tan group (based on the Model group treatment, the cells were treated with high‐dose Tan) following treatment with tanshinone IIA and miR‐34a‐5p group (based on Model group treatment, the cells were transfected with miR‐34a‐5p mimics) (*p* < .001, respectively, Figure 13a,b). A significant decrease in the rate of cell proliferation and a significant increase in the rate of cell apoptosis were observed in the Tan + si‐miR‐34a‐5p group following si‐miR‐34a‐5p transfection (*p* < .001, respectively, Figure 13a,b).

### 3.14 Effects of miR‐34a‐5p on associated proteins

**FIGURE 14:**
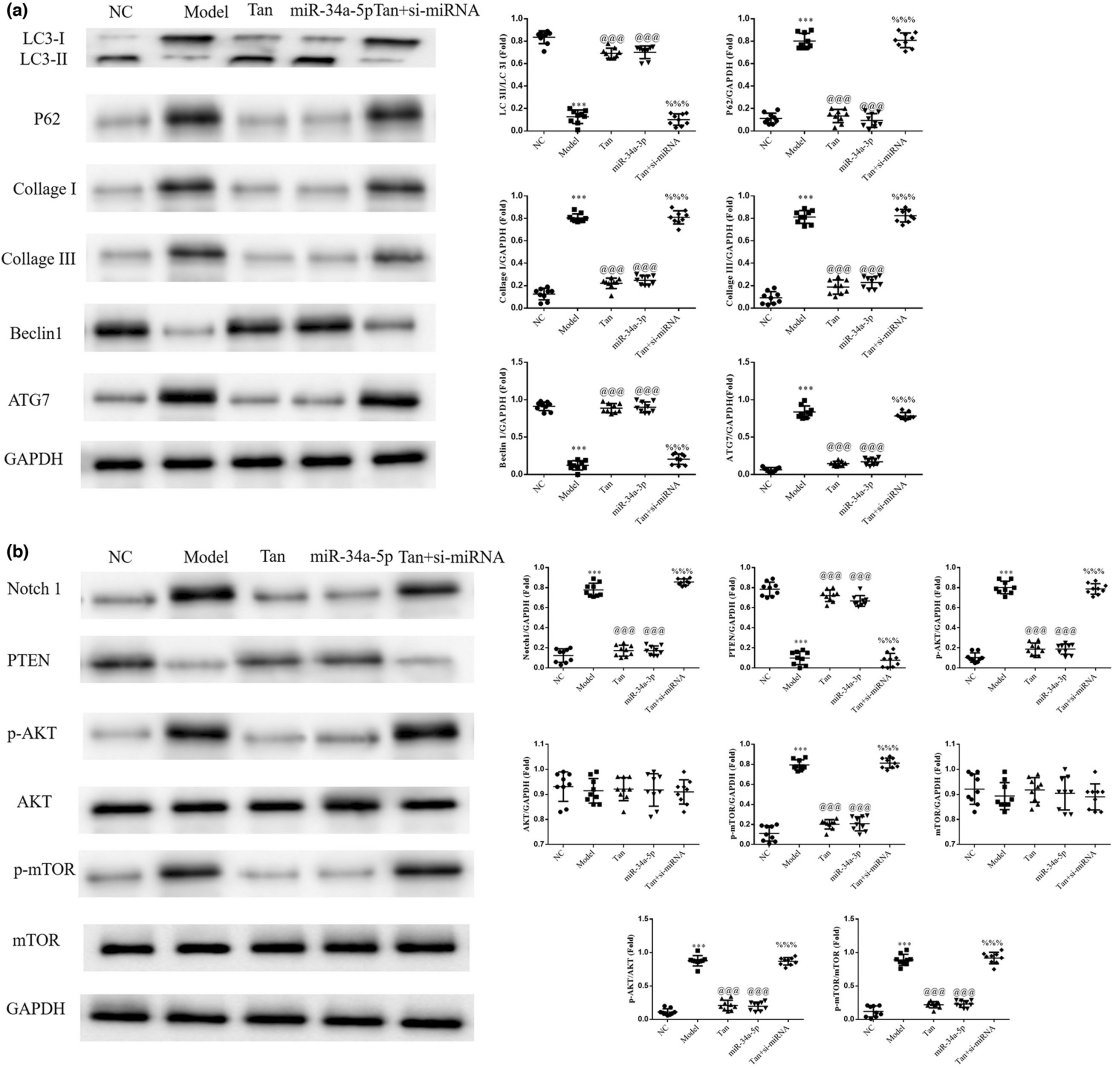
miR‐34a‐5p affects relative protein expression. The following groups were established: NC, HK‐2 cells treated with normal; Model, HK‐2 cells treated with high concentrations of glucose; Tan, HK‐2 cells treated with 25 mg/L tanshinone IIA in high concentrations of glucose; miR‐34a‐5p: HK‐2 cells transfected with miR‐34a‐5p mimics in high concentrations of glucose; Tan + si‐miRNA, HK‐2 cells transfected with si‐miR‐34a‐5p and treated with 25 mg/L tanshinone IIA in high concentrations of glucose. Protein expression levels of (a) LC3, Beclin1, ATG7, P62, Collagen I, and Collagen III and (b) Notch1, PTEN, p‐AKT, and p‐mTOR were determined using western blotting. ^***^ *p* < .001 vs. NC group; ^@@@^ *p* < .001 vs. Model group; ^%%%^ *p* < .001 vs. Tan group. miRNA/miR, microRNA; NC, negative control; Notch1, notch receptor 1; p‐, phosphorylated.

The results of the western blot analysis revealed a decrease in the LC3II/LC3I ratio and Beclin1 protein and a significant increase in the protein expression levels of P62, ATG7, Collagen I, Collagen III, Notch1, PTEN, p‐AKT, and p‐mTOR in the Model group, compared with the NC group (*p* < .001, respectively, Figure 14a,b). Furthermore, following treatment with tanshinone IIA or miR‐34a‐5p mimics transfection, the Tan group demonstrated an increase in the LC3II/LC3I ratio and Beclin1 protein, and a marked decrease in the protein expression levels of P62, ATG7, Collagen I, Collagen III, Notch1, PTEN, p‐AKT, and p‐mTOR (*p* < .001, respectively, Figure 14a,b). In addition, a notable decrease in the LC3II/LC3I ratio and Beclin1 protien, and a marked increase in the protein expression levels of P62, ATG7, Collagen I, Collagen III, Notch1, PTEN, p‐AKT, and p‐mTOR were observed in the Tan + si‐miRNA group following transfection with si‐miR‐34a‐5p, compared with the Tan group (*p* < .001, respectively, Figure 14a,b).

### 3.15 Detection of relevant gene expression using RT‐qPCR

**FIGURE 15:**
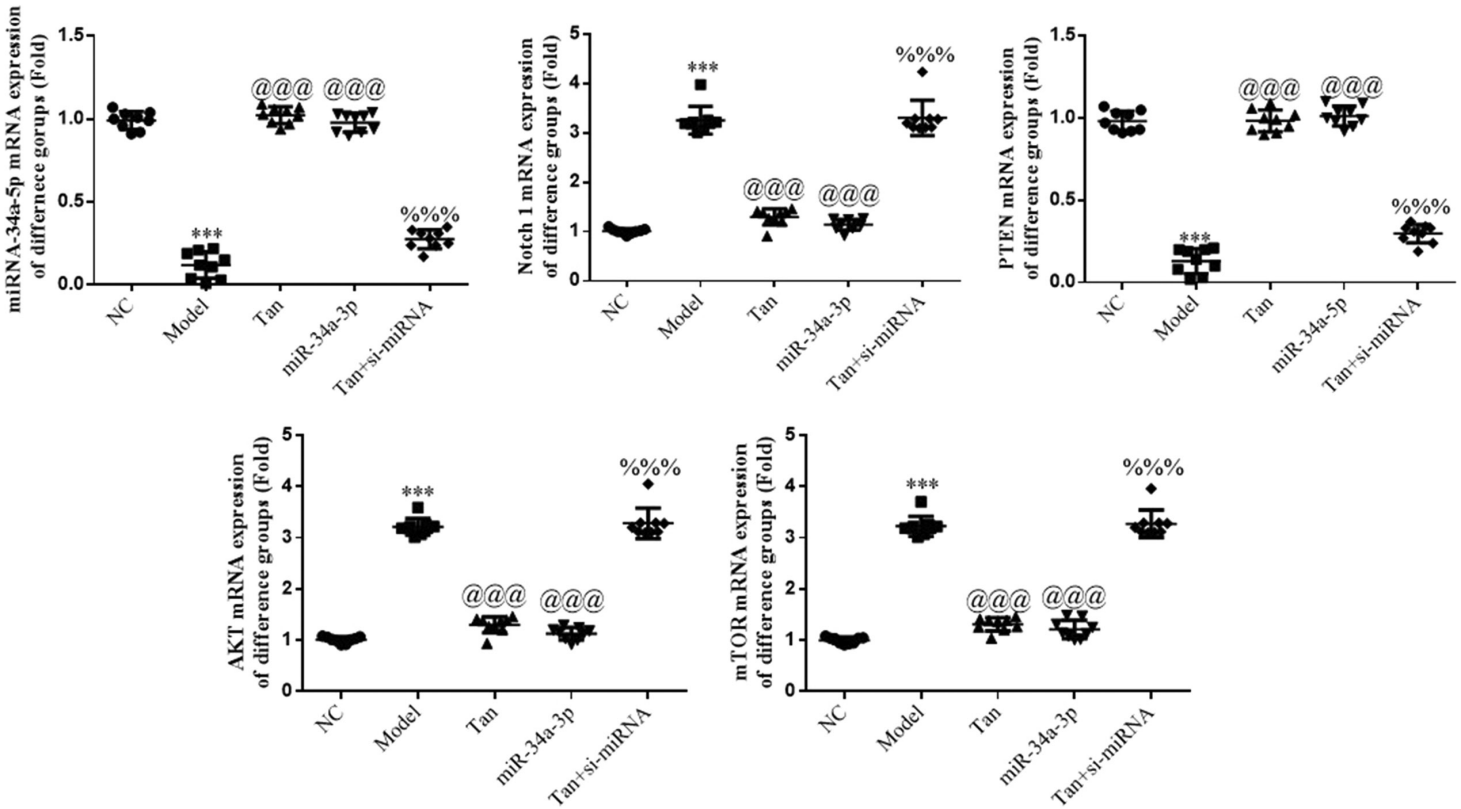
Gene expression determined using reverse transcription‐quantitative PCR assays. The following groups were established: NC, HK‐2 cells treated with normal; Model, HK‐2 cells treated with high concentrations of glucose; Tan, HK‐2 cells treated with 25 mg/L tanshinone IIA in high concentrations of glucose; miR‐34a‐5p: HK‐2 cells transfected with miR‐34a‐5p mimics in high concentrations of glucose; Tan + si‐miRNA, HK‐2 cells transfected with si‐miR‐34a‐5p and treated with 25 mg/L tanshinone IIA in high concentrations of glucose. ^***^ *p* < .001 vs. NC group; ^@@@^ *p* < .001 vs. Model group; ^%%%^ *p* < .001 vs. Tan group. miRNA/miR, microRNA; NC, negative control.

The results of the RT‐qPCR analysis revealed a decrease in the gene expression levels of miR‐34a‐5p and PTEN, and a significant increase in the gene expression levels of Notch1, AKT, and mTOR in the Model group, compared with the NC group (*p* < .001, respectively, Figure 15). Following treatment with tanshinone IIA or miR‐34a‐5p mimics transfection, an increase in the gene expression levels of miR‐34a‐5p and PTEN, and a decrease in the gene expression levels of Notch1, AKT, and mTOR were observed in the Tan group, compared with the Model group (*p* < .001, respectively, Figure 15). Moreover, following transfection with si‐miR‐34a‐5p, a decrease in the gene expression levels of miR‐34a‐5p and PTEN, and an increase in the gene expression levels of Notch1, AKT, and mTOR were observed in the Tan + si‐miRNA group (*p* < .001, respectively, Figure 15).

### 3.16 Effects of miR‐34a‐5p on the protein expression levels of LC3B and P62

**FIGURE 16:**
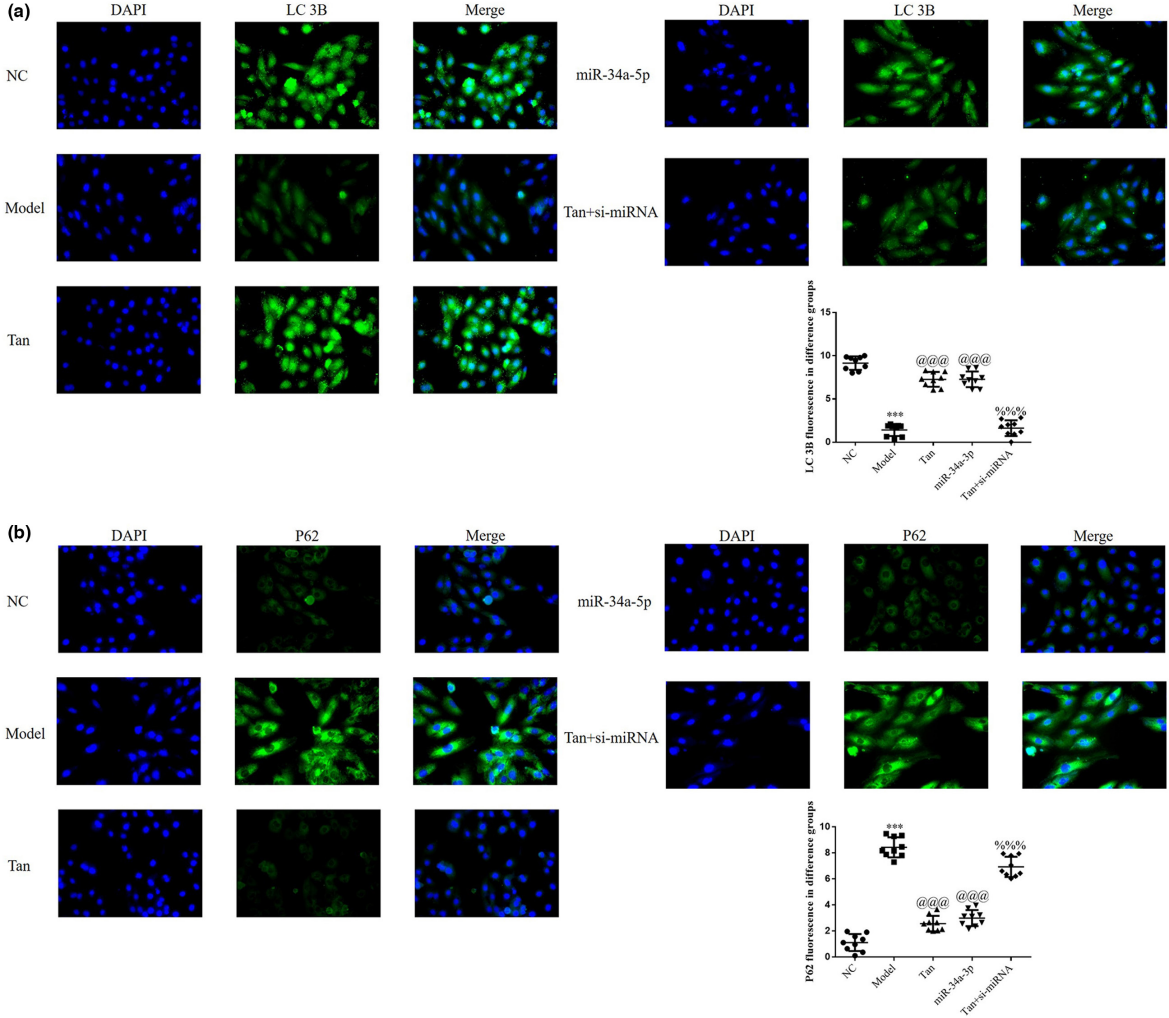
LC3B and P62 protein expression levels determined via cell immunofluorescence. The following groups were established: NC, HK‐2 cells treated with normal; Model, HK‐2 cells treated with high concentrations of glucose; miR‐34a‐5p: HK‐2 cells transfected with miR‐34a‐5p mimics in high concentrations of glucose; Tan, HK‐2 cells treated with 25 mg/L tanshinone IIA in high concentrations of glucose; Tan + si‐miRNA, HK‐2 cells transfected with si‐miR‐34a‐5p and treated with 25 mg/L tanshinone IIA in high concentrations of glucose. (a) LC3B and (b) P62 protein expression levels were determined via cell immunofluorescence (magnification, ×200). ^***^ *p* < .001 vs. NC group; ^@@@^ *p* < .001 vs. Model group; ^%%%^ *p* < .001 vs. Tan group. miRNA/miR, microRNA; NC, negative control.

The results of the immunofluorescence assay demonstrated a decrease in the protein expression level of LC3B, and an increase in the protein expression level of P62 in the Model group, compared with the NC group (*p* < .001, respectively, Figure 16a,b). Following treatment with tanshinone IIA or miR‐34a‐5p mimics transfection, an increase in the protein expression level of LC3B and a significant decrease in the protein expression level of P62 were observed in the Tan group (*p* < .001, respectively, Figure 16a,b). In addition, a decrease in the protein expression level of LC3B and an increase in the protein expression level of P62 were observed in the Tan + si‐miRNA group following transfection with si‐miR‐34a‐5p, compared with the Tan group (*p* < .001, respectively, Figure 16a,b).

### 3.17 Association between miR‐34a‐5p and Notch1

**FIGURE 17:**
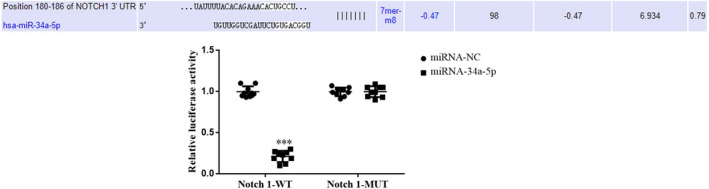
Correlation analysis between miR‐34a‐5p and Notch1. ^***^ *p* < .001 vs. miRNA‐NC group. miRNA/miR, microRNA; NC, negative control; Notch1, notch receptor 1.

In Notch1‐mutant HK‐2 cells, no significant difference was observed in the immunofluorescence intensity in the miR‐34a‐5p group compared with the miRNA‐NC group. Moreover, in Notch1‐wild‐type HK‐2 cells, the fluorescence intensity of the miR‐34a‐5p group was significantly reduced, compared with the miRNA‐NC group (*p* < .001, Figure 17). The results of the present study demonstrated the role of miR‐34a‐5p in the regulation of Notch1 expression in HK‐2 cells.

## 4 DISCUSSION

Diabetic nephropathy (DN) is one of the most common complications in patients with DM. Microalbuminuria is an early sign of DN in patients with DM, which may indicate the occurrence of renal lesions. Further associated pathological manifestations are glomerular hypertrophy, increased extracellular matrix production, glomerulosclerosis, interstitial fibrosis, and multiple lesions in other areas (Chevalier et al., 2009; Yanfang et al., 2013). The Notch signaling pathway is a key conserved transmembrane signal transduction pathway that determines cell fate. In mammals, the pathway is composed of four receptors (Notch1, Notch2, Notch3, and Notch4), classical ligands and nonclassical ligands (Radtke et al., 2010). Among them, Notch1 plays an essential role in maintaining the function of renal tubular epithelial cells (Mukherjee et al., 2019). Moreover, the Notch signaling pathway further exacerbates renal fibrosis of DN by aggravating oxidative stress (Han et al., 2017). Tian et al. (2018) demonstrated that the activation of Notch promoted the epithelial–mesenchymal transdifferentiation of renal tubular epithelial cells and stimulated the process of renal fibrosis in DN. Thus, Notch may participate in the progression of fibrosis in DN through a number of mechanisms.

It has previously been established that the Notch signaling pathway and autophagy regulate podocyte differentiation. Under an abnormally activated Notch signaling pathway, upregulation in autophagy levels reduces podocyte differentiation, alleviates podocyte injury, and relieves renal dysfunction (He et al., 2018; Zhang et al., 2017). In addition, the results of a previous study demonstrated that in a high‐glucose environment, the activation of Notch1 damaged podocytes, destroyed the balance between apoptosis and autophagy, thus producing a negative effect on the functional recovery of podocytes. Therefore, activation of the Notch signaling pathway in the kidney may lead to podocyte dysfunction through disruption of the levels of autophagy.

Autophagy is an evolutionarily conserved trait in which damaged organelles and intracellular misfolded proteins are degraded by lysosomes, cytotoxic protein aggregates are eliminated, and cells have the ability to recycle energy from mitochondria in order to maintain cell survival (Johansen & Lamark, 2011). Thus, the association between autophagy and renal diseases has become a primary research focus. Normal autophagy plays a key role in maintaining cell function, whereas abnormal autophagy results in cell dysfunction. It has previously been demonstrated that renal interstitial fibrosis is one of the primary pathological features in the development of DN. Notably, autophagy is also involved in this process (Choi, 2020). Furthermore, the mTOR signaling pathway is an essential mechanism for the negative regulation of autophagy (Lenoir et al., 2016). Inhibition of mTOR promoted autophagy, while the reactivation of mTOR, as a key feedback mechanism, further inhibited autophagy and initiated lysosomal remodeling (Kang et al., 2011).

In the present study, decreased LC3B protein expression levels and significantly increased P62, p‐AKT, and p‐mTOR protein expression levels were observed in DN mice and HK‐2 cell models, accompanied by inhibited autophagy, activation of the AKT/mTOR signaling pathway, and deposition of Collagen I and Collagen III. However, a notable improvement was observed following treatment with tanshinone IIA. Furthermore, the results of the present study revealed that 25 mg/kg tanshinone IIA exhibited beneficial therapeutic effects on DN‐induced renal fibrosis. In addition, the AKT/mTOR signaling pathway exerts a regulatory role in the process of autophagy (Wang et al., 2018). Among these proteins, PTEN is the only tumor suppressor gene found to have both lipid phosphatase activity and protein phosphatase activity (Li et al., 1997). PTEN suppresses tumors and mediates the cell signal transduction pathway. PTEN also negatively regulates the PI3K/AKT signaling pathway as a lipid phosphatase (Gao et al., 2018; Tao et al., 2019; Ye et al., 2017). The role of Notch1 in tubulointerstitial fibrosis in DN was verified in the present study. The results highlighted a decrease of PTEN protein expression in DN animal and cell models, and Notch1 exhibited a negative association with PTEN. Notch1 activation of the AKT/mTOR pathway subsequently suppressed autophagy, leading to the occurrence and development of renal fibrosis. However, the results of the present study indicated an inhibited activation of Notch1 and an increase in the expression levels of PTEN following treatment with tanshinone IIA (tanshinone IIA has no synergistic effect on Notch 1 inhibitor, the data are shown in Figures 9, 10, 11, 12), which further decreased the activity of the AKT/mTOR pathway, thus promoting autophagy (with autophagy inhibitor, which was an mTOR agonist, intervening, tanshinone IIA's treatment effects disappeared, the data are shown in Figures 9, 10, 11, 12) and improving renal fibrosis. Further studies are required to determine the specific mechanisms underlying tanshinone IIA in Notch1 regulation.

The miRNAs are the primary focus in a number of studies. The results of previous studies have indicated that miRNAs play an important role in the occurrence and development of chronic kidney disease (Cheng et al., 2020; Fujii et al., 2019; Klimczak‐Tomaniak et al., 2020; Shen et al., 2020). The results of the present study demonstrated a significant decrease in the expression levels of miR‐34a‐5p in renal tissues and cells of the Model group, suggesting that a reduced expression of miR‐34a‐5p may play a role in the development of renal fibrosis. Moreover, treatment with tanshinone IIA resulted in an increase in the expression level of miR‐34a‐5p. Furthermore, the positive therapeutic effects of tanshinone IIA on renal fibrosis were reversed following knockdown of miR‐34a‐5p. The results of the dual‐luciferase reporter assay verified that miR‐34a‐5p has the ability to target Notch1 in HK‐2 cells. Thus, it was suggested that tanshinone IIA may exert a beneficial role in improving renal fibrosis by upregulating the expression of miR‐34a‐5p and directly targeting Notch1.

There were some limits in our study, we just discuss the effects of tanshinone IIA on improve DN‐induced renal tubular injury, however, the effects of tanshinone IIA on glomerular injury in DN. We used TUNEL and flow cytometry to evaluate the apoptosis cell number and rate in kidney tissues or HK‐2 cell lines of different groups, and did not measure apoptosis by other ways like as measuring caspase‐3 and caspase‐9 protein expressions. And we did not discuss miR‐34a‐5p's effects on tanshinone IIA treatment in vivo study. In our future study, we will discuss those limits and continue to relative study.

In conclusion, our research found that tanshinone IIA had improved DN‐induced renal tubular injury via miR‐34‐5p/Notch1 axis which was in correlation with autophagy in vitro and in vivo study.

## CONFLICT OF INTEREST

The authors declare no conflict of interest.

## ETHICAL STATEMENT

This study was approved by the Ethics Committee of The Second People's Hospital of Hefei.
